# Granting analysis of the credit financing of the platform-type highway transportation supply chain

**DOI:** 10.1371/journal.pone.0303807

**Published:** 2024-07-10

**Authors:** Lili Xu, Feng Liu, Xuejian Chu

**Affiliations:** 1 Business School, Shanghai Dianji University, Shanghai China; 2 School of Management, Shanghai University, Shanghai China; University of Cagliari: Universita degli Studi Di Cagliari, ITALY

## Abstract

Against the background of digital development, this study’s research object is the platform-based highway transportation supply chain. It also analyzes two modes of supply chain financial credit financing, namely, upstream, and downstream enterprises of the platform, and network freight platform as the main financing body. Notably, the financial provider sets up a transaction credit based on the principle of business truth, and closed-loop transactions, determine the upper limit of the credit line based on the principle of financing self-compensation, build the expected profit maximization model, and establish the optimal credit line. Combined with the Highway Freight Index and Logistics Prosperity Index, the dynamic early warning value is established for the financing mode, where the platform as the main financing body. Through numerical analysis, the credit line and expected profit increase with the transaction credit, expected freight volume, and credit interest rate under the two modes, and the increase deriving from the credit interest rate is more significant. Finally, this paper describes the two-dimensional credit matrix of the financing subject via transaction credit and credit interest rate, which provides an intuitive credit reference for financial institutions to conduct the credit financing of the platform-based highway transportation supply chain.

## 1. Introduction

China has a huge highway transportation market. In 2021, the total cost of domestic social logistics is 16.7 trillion-yuan, accounting for 14.6% of the GDP. The total transportation cost is 9 trillion yuan, where of the scale of highway freight exceeds 7 trillion-yuan, accounting for 78% of the total^.^ However, as many as 2,737,400 small and micro highway transport enterprises exist in China, accounting for 84.52% of the national highway transportation, with an industry concentration of only 1.2%. Meanwhile, 83.7% of the enterprises have meager incomes or even incur losses. On average, each enterprise owns only approximately 1.5 vehicles, and the funding gap exceeds 3 trillion yuan per year [1]. The huge transportation demand of the "large transportation capacity market" requires a certain number of enterprises to bear, unlike "small transportation enterprises" due to the weak capital advance capacity and limited carrying capacity, thereby resulting in the development contradiction between the large market and small enterprises, with the latter having a large capital gap and strong financing demand. However, small and micro highway capacity logistics enterprises are unable to obtain bank financing due to their small scale, asset-light strategy, lack of the perfect three financial statements and sufficient collateral, lack of subject credit, and credit loans such as accounts receivable factoring. Only Logistics freight prepaid, with an annual financing demand of about 600 billion yuan, but less than 5% of loans from banks. However, the characteristics of low service quality of transportation capacity, frequent collusion and hidden behaviors, high moral hazard, as well as operational risk [2], abstraction, invisibility and subcontracting at different levels hinder transportation supply chain financing in the actual development process, and small and micro highway transportation capacity logistics enterprises are deeply mired in financing difficulties.

With the development of the network freight platform, a batch "Internet Plus" road freight platform enterprises have emerged in China. They integrate logistics resources with the help of Internet technology and platform economy thought, and strive to solve contemporary road freight problems [3]. The road freight platform has a strong ability to not only aggregate information resources [4], but also gather many small and micro homogeneous highway capacity logistics enterprise resources, and it has the technology of Internet Plus logistics operations to meet the supervision demands of highway transportation supply chain finance. Financial institutions recognize financing enterprises based on trust in network freight platform enterprises. By breaking the information barriers between banks and enterprises and improving the willingness of financial institutions to grant credit, the financing availability for small and micro highway capacity logistics enterprises can be increased.

**Fig 1:**
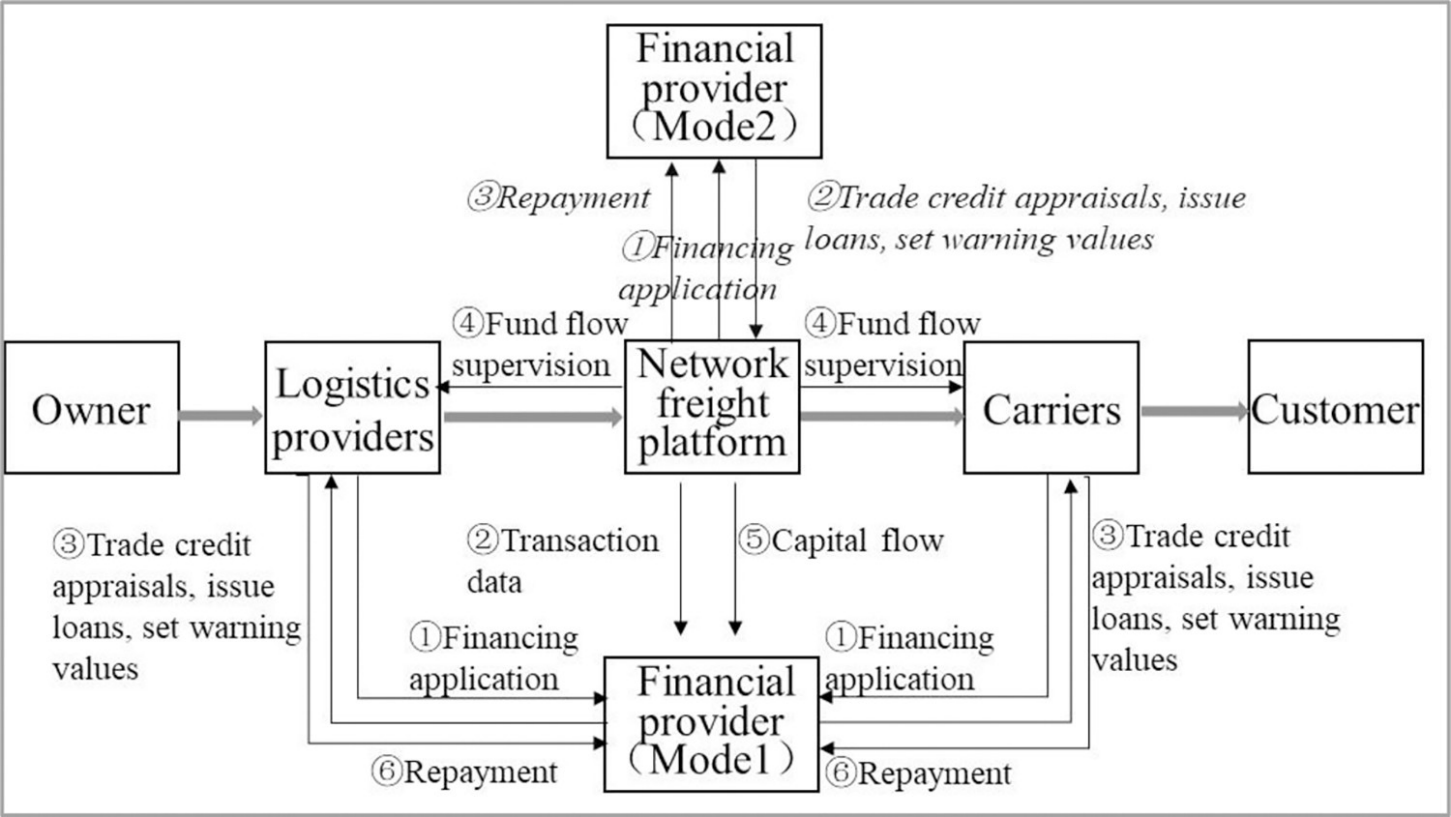
Platform-based highway transportation supply chain and credit process.

A highway transportation capacity supply chain based on the network freight platform, namely, the platform-based highway transport capacity supply chain, includes five main bodies: owner, logistics provider, network freight platform, carrier, and customer. Among them, the owner refers to the enterprise with the ownership of the goods and demands for freight transportation. Logistics providers mainly refer to third-party logistics enterprises. Based on perfect qualifications and mature operating abilities, third-party logistics enterprises often have stable owner resources. The network freight platform has strong information technology capabilities and participates in the entire service process, including credit assessment, vehicle scheduling tracking, payment, and settlement, etc. Simultaneously, contracts, documents, bills, and so on, are digitized through blockchain and other technologies to realize visual and transparent supervision, and they have data analysis capabilities for vehicle sources and goods sources. The network freight platform determines the platform service income based on the difference between the contract amount collected from the logistics provider and the contract finance paid to the carrier and it bears the risk of goods loss or damage during transportation; however, the risk amount generally does not exceed the freight amount. Carriers refer to enterprises that undertake the actual transportation of goods, such as special lines, motorcades and drivers, the latter two of which often do not pay for goods in advance. The customer is the recipient of the goods and pays the corresponding freight according to the contract upon confirmation of the goods. On the one hand, the traditional supply chain relies on individual trust among participants rather than being primarily based on the credit system. On the other hand, the platform-based highway transportation supply chain breaks the individual closure and establishes a credit system for the entire chain. There are two main modes of credit granting in a platform-based highway transportation supply chain (as shown in Fig 1):

(1) Take the platform’s upstream and downstream enterprises as the main financing body, that is, logistics providers or carriers. Under this mode, first, logistics providers or carriers submit corresponding materials to financial providers for financing application; second, the network freight platform opens the transaction data of corresponding financing entities to financiers, including contracts, orders, waybills, receipts, statements and invoices; third, The financial provider evaluates the transaction credit (true business and closed-loop transaction) of the financing enterprise according to the transaction data, determines the quota, and issues loans based on the evaluation results; fourth, the network freight platform supervises the flow of funds for issuing loans; fifth, the results are reported to financiers in real time; and sixth, the financing enterprise shall repay the loan when it is due.

(2) Take the network freight platform as the main financing body and carry out unified credit granting. This mode is relatively simple in process, that is, first, the network freight platform applies for financing from financiers; second, financial providers evaluate the transaction credit of the network freight platform, determine the credit line, issue loans, and set dynamic early warning values according to the future repayment ability of the network freight platform; and third, repayment due from the network freight platform.

However, the determination of the credit line according to platform-based highway capacity supply chain credit financing, the factors that affect the credit line, and whether the credit line determination methods differ under different credit models is essential. These problems are related to whether platform-based highway transportation supply chain credit financing can be implemented, whether the financial model can be innovated, and whether supply chain financing can solve the financing difficulties and expensive financing of small and medium-sized enterprises

Therefore, based on the transaction data of small and medium-sized micro-logistics enterprises gathered by the network freight platform, this study intends to confirm the basic transaction links substantially by returning to the origin of transaction authenticity to realize transaction credit enhancement and carry out credit financing of the highway transportation supply chain finance. Based on the principles of real business, closed-loop transactions, and financing self-compensation, the quantitative index is formed, the profit maximization model of financial institutions is constructed, and the credit line analysis method for highway transportation supply chain credit financing based on the network freight platform is provided. Simultaneously, based on the characteristics of the network freight platform with data assets as the core and the principle of data reaching and price marking, the early warning value is further set to hedge the credit risk that may be brought about by transaction credit enhancement, present financial institutions with quantitative supervision after the credit granting of platform-based highway transportation supply chain credit financing, and provide methods and countermeasures.

## 2. Materials and methods

### 2.1 Review of related research

Currently, the research on the credit financing of platform-based supply chain mainly focuses on three aspects.

The first aspect primarily entails the commodity supply chain constructed by e-commerce platforms and involves research on supply chain finance. Qinglie Wu, Jin Wang and Zhong Wu take electronic orders as an example to study B2B supply chain financing and find that B2B platform-led supply chain finance is superior to bank-led unsecured supply chain finance [5]. Chengfu Wang, Xiaojun Fan and Zhe Yin divide e-commerce platforms into two forms, namely, active and conservative financing, based on the different degrees of integration of the lending business and conclude that active e-commerce platform financing can realize the coordination of supply chain finance, while conservative financing may not be realized [6]. Li Xiaoli and Cheng Shuo introduce the network effect of e-commerce platforms into the cooperation process of the supply chain finance business, pointing out that e-commerce platforms will choose the cooperation strategy at a lower level of revenue sharing, and banks do not need supervision to realize complementary advantages and the rational allocation of resources [7]. Liu, L., Peng, Q. point out that when the amount of financing that the e-commerce platform needs to provide is too high, the platform will refuse green financing, and when the amount of financing is low, it is more likely to realize financing [8]. Xiaoyu Wang and Fasheng Xu examine the value of smart contracts in trade financing [9]. Nina Yan and Xiuli He study the impact of the trade credit plan on financing and operational decisions based on income, bankruptcy cost and service level [10]. Although these forms of research focus on platforms engaged in supply chain finance, their primary focus is on commodity supply chains. The highway transportation supply chain has a layer upon layer subcontracting relationship from the top cargo owner to the service integrators such as large third-party logistics, then to the service intermediaries such as small third-party logistics and special lines, and finally to the service providers such as fleets and drivers. The traditional credit granting objects and credit granting scenarios for the commodity supply chain are no longer applicable.

The second aspect is to study the participant game around the network freight platform. Based on evolutionary game theory, Pang Ruiqi et al. take traditional logistics supervision and digital trust supervision as the strategic space of the network freight platform, and default and compliance as the strategic space of small and micro transportation enterprises. Based on evolutionary game theory, they build a dynamic game model between the network freight platform and small and micro transportation enterprises and discuss the impact of the choice of the platform financing supervision strategy on the financing credit risk of small and micro transportation enterprises [1]. Gui Yunmiao et al. establish the game decision model for an oligopoly competitive platform based on Hotelling and discuss the pricing strategy of the network freight platform from four different user ownership perspectives [11]. Guo Xiaolong et al. build a tripartite game model including manufacturers, network freight platforms and retailers and study logistics service effort level decisions and supply chain pricing strategies under different service modes [12]. Deng Shuai et al. establish a three-player evolutionary game model to analyze the interactions among freight carriers, shippers, and logistics platforms, as well as the asymptotic equilibrium and evolutionary stability strategies of the three-player game [13]. Although this kind of research focuses on the highway transportation supply chain dominated by the network freight platform, it considers the participant game, carries out risk analysis or platform pricing through the game, and the characteristics of the transportation supply chain and the lack of collateral in small and micro highway transportation enterprises have not been combined. It also rarely combines with supply chain finance to solve the financing problems of small and medium-sized micro-transportation enterprises. Even when combined with supply chain finance, the focus remains on reducing bank credit risk rather than on specific credit lines.

The third aspect involves conducting innovative research on digital credit and credit creation in connection to platform-based supply chain finance. Feng Bao and Zhang Zuomin Yang compare and study the tripartite evolutionary game model of core enterprises, small and medium-sized enterprises, and financial institutions in platform-type supply chain finance before and after the introduction of blockchain. After the introduction of blockchain, platform-type core enterprises have changed from evaluating the qualifications and pledges of small and medium-sized enterprises to more efficient digital financing in the chain, which has relaxed the conditions for the financing game system to reach the Pareto optimal state, and the supply chain finance financing has been Pareto improved [14]. Dong Li and Chen Jinlong note that platform-type supply chain finance has a credit creation effect and analyzed the credit creation process and sensitive factors of supply chain finance platforms using money multipliers in game theory and money supply theory. It is pointed out that the initial credit bills (digital bills) issued by core enterprises to suppliers are used instead of currencies, which are split and transferred layer by layer, thus realizing the credit derivation function. The credit creation ability is related to the initial credit amount opened by core enterprises, credit circulation speed, credit settlement period, supplier level, bank credit line to core enterprises, credit bill discount ratio and holding maturity ratio [15]. Zhan Yongzhi et al. innovatively combine the reciprocal psychological utility and material utility of suppliers, taking providing reverse factoring and extending the credit period as their reciprocal conditions respectively, and defined the reciprocal psychological and material utility conditions for suppliers to accept the requirements of core enterprises to extend the credit period, hence providing a direct decision-making basis for core enterprises to reasonably determine the extension period of the credit period and balance interests [16]. Although this type of research is not combined with the network freight platform, its innovative credit concept provides insight and reference for this study to use contracts, documents, and other information carriers to observe the transaction process of the platform-type highway transportation supply chain, realize the transparency and authenticity of information, and transform transaction credit into financial credit, thus realizing traditional credit enhancement.

Therefore, this study intends to take the highway transportation supply chain as the research scope, focus on the platform-type highway transportation supply chain, take credit financing as the main financing mode, take credit line as the research object, and study the platform-type highway transportation supply chain credit financing by combining model construction with numerical analysis, which has the following innovations:

(1) Research perspective innovation. Different from the platform-type commodity supply chain which takes the pledge of goods rights as the financing object, it only focuses on the advantages of the platform. This study takes the credit system of the whole chain constructed by contracts, orders, waybills, receipts, statements and invoices in the platform-type highway transportation supply chain as the financing object, focuses on the credit decision problem, and provides a new research perspective for solving the financing problems of small, medium and micro transportation enterprises.

(2) Research theoretical innovation. On the one hand, this study describes the structure of the platform-type highway transportation supply chain, and expounds two credit financing models with the upstream and downstream enterprises of the platform as the financing subject and the platform as the financing subject, expanding the theoretical basis of the platform-type highway transportation supply chain. On the other hand, this study takes business as the basis for credit granting, forms transaction credit through the data of small, medium and micro transportation enterprises gathered by the network freight platform, takes the credit extension exposure of financial institutions covered by the self-compensation of business income as the judgment of the credit line, builds a profit maximization model and sets a dynamic early warning value, and enriches the literature research system of platform-type highway transportation supply chain finance.

(3) Innovation of research methods. This study combines the methods of discretionary cash flow, probability density and regression analysis, visualizes the transaction credit, constructs a credit line model from different perspectives, and establishes a two-dimensional credit matrix, which provides a reference for the determination of credit line for financial institutions. At the same time, through the numerical analysis of the impact of transaction credit, freight volume, credit rate on the credit line, the obtained results are completely consistent with the model, and the closed-loop verification of the whole study is realized.

### 2.2 Problem description

Platform-type highway transportation supply chain credit financing focuses on the business itself, with the principle of “business truth” and “closed-loop transaction.” Business truth is the real freight transaction contract of the transportation supply chain enterprise, and the documents are complete, coherent, standardized, and effective, while the freight track control is accurate, freight process operation is standardized, and capital flow is clear. The closed-loop transaction means that contracts, documents, invoices, and so on, can be compared and verified in all links of the supply chain of the financing enterprise, forming a complete transaction logic. To this end, business data such as contracts, orders, waybills, receipts, statements, and invoices, are gathered by the network freight platform with the platform’s own technology, capability, and reputation to evaluate the transaction credit of financing enterprises and form credit access conditions. Based on the principle of “financing self-compensation,” the future business income corresponding to the financing enterprise is the repayment source of its credit funds, and the upper limit of credit line is established. Based on the principle of “controllable risks,” the capital flow and repayment ability are supervised after credit granting, and dynamic early warning values are set. According to the above-mentioned principles, this study constructs the credit line model, determines the optimal credit line, and sets the dynamic early warning value for two modes: “taking the upstream and downstream enterprises of the platform as the financing subject" and "taking the network freight platform as the financing subject.”

Based on the credit and follow-up models, this study puts forward the following assumptions:

(1) Since the network freight platform was piloted with the concept of a car-free carrier in 2016, listed companies such as LOGORY and YMM have appeared. According to the Guide to Road Freight Transportation Operation and Service of Network Platform, the platform must perform eight functions: information release, whole-process monitoring, online transaction, financial payment, online evaluation, consultation and complaint, inquiry and statistics, and data retrieval. Therefore, the transaction credit evaluation index can be established from the data integrity, data authenticity, data security, operation efficiency, standardized operation, and service quality of the platform [17], and the transaction credit *k*_1_,0<*k*_1_≤1, can be determined.

(2) Future business income is used as the repayment source of credit funds, and discretionary cash flow is usually formed after deducting the corresponding costs as a direct repayment guarantee. According to the investigation of risk management and control measures of leading enterprises, such as Far East Horizon Limited, its discretionary cash flow = initial monetary funds of financing enterprises + operating net cash flow-rigid expenditures, such as bank loans and financial leasing. Among them, operating net cash flow = net profit of financing enterprise + depreciation and amortization + non-capitalized financial expenses-non-recurring gains and losses (investment income, etc.).

(3) Compared to a single enterprise in the highway transportation supply chain, the network freight platform gathers many enterprises, and its future business income benefits from the contract amount formed by the convergence of many supply chain enterprises on the platform, which is affected more by the overall development of the logistics industry than by a single enterprise. The Logistics Prosperity Index (LPI) consists of 12 sub-indices, including total business volume and expected business activities, which reflects the overall development and operation of the logistics industry [18]. The prosperity of the logistics industry has a significant impact on the development of the industrial economy in the next 3 ~ 6 periods [19], and the prosperity index of the logistics industry is the Granger cause of producer price index [20]. The Highway Freight Index (HFI) is a measure of the fluctuation in the highway transportation market, and it has an important predictive function for China’s highway transportation industry [21]. Therefore, this study assumes a linear correlation between the future business income of the network freight platform and LPI and HFI.

(4) The logistics service demand of the highway transportation supply chain based on the network freight platform differs from to business contract logistics, which has random uncertainty. According to Honglin Yu et al.^,^ the random demand obeys normal distribution [22].

(5) As risk-neutral enterprises, financial institutions aim to maximize profits.

(6) The financing enterprise is assumed to have limited liability, and the debt repayment liability comes from the future business income. The financing enterprise repays the principal and interest in equal amounts, and the repayment amount is evenly distributed.

### 2.3 Symbol description

To clearly understand the relevant parameters and symbols in this study, they are summarized and explained in Table 1.

**Table 1:** Description of parameters and symbols.

Parameter category	Parameter symbol	Parameter interpretation
**Decision variable**	*L*_*i*_(*i* = 1,2,)	*i* = 1 Credit line of financial provider to logistics provider/carrier, and credit line of financial provider to network freight platform *i* = 2
**parameter**	*k*_*i*_(*i* = 1,2,)	*i* = 1 Transaction credit of logistics provider/carrier and transaction credit of network freight platform *i* = 2
	*q*_*i*_(*i* = 1,2⋯*m*)	Expected freight volume of the I-th enterprise within T time (unit: ton)
	*p*	Unit freight rate (unit: yuan/ton km)
	*d*	Transportation distance (unit: km)
	*S*	Logistics/Carrier Freight Service Revenue
	*S*_*p*_	Network freight platform services revenue
	*θ*_*i*_(*i* = 1,2,)	*i* = 1 Net profit rate of logistics provider/carrier and net profit rate of network freight platform *i* = 2
	*γ*_*i*_(*i* = 1,2,)	*i* = 1 Depreciation and amortization of logistics providers/carriers, depreciation, and amortization of network freight platform *i* = 2
	*F*_*0i*_(*i* = 1,2,)	*i* = 1 beginning monetary funds of logistics providers/carriers and beginning monetary funds of network freight platform *i* = 2
	*F*_1*i*_(*i* = 1,2,)	*i* = 1Non-capitalized financial expenses of logistics providers/carriers and non-capitalized financial expenses of network freight platforms *i* = 2
	*F*_2*i*_(*i* = 1,2,)	*i* = 1 Non-operating profit and loss of logistics providers/carriers and non-operating profit and loss of network freight platforms *i* = 2
	*e*_*i*_(*i* = 1,2,)	*i* = 1 Rigid expenditures such as bank loans and financial leases of logistics providers/carriers *i* = 2 Rigid expenditures such as platform commercial bank loans and financial leases
	${F^{*}}_{i}(i=1,2)$	*i* = 1 Net operating cash flow of logistics providers/carriers and net operating cash flow of network freight platform *i* = 2
	*F*_*i*_(*i* = 1,2,)	*i* = 1 Logistics providers/carriers can freely dispose of funds, and network freight platform can freely dispose of funds *i* = 2
	*μ*_*i*_(*i* = 1,2⋯*m*)	Expected value of the i-th enterprise’s freight volume
	*σ*_*i*_(*i* = 1,2⋯*m*)	Standard deviation of freight volume for the i-th enterprise
	*x*_*i*_(*i* = 1,2,)	*i* = 1 Repayment amount of logistics provider/carrier and repayment amount of network freight platform *i* = 2
	*r*_*i*_(*i* = 1,2,)	*i* = 1 Financing interest rate granted by financial provider to logistics providers/carriers *i* = 2 Financing interest rate granted by financial provider to network freight platform
	*c*_*i*_(*i* = 1,2,)	*i* = 1 Cost of credit financing from financial provider to logistics providers/carriers *i* = 2 Cost of credit financing from financial provider to network freight platform
	*I*_*p*_	Net profit of the network freight platform
	*T*	Credit period of financial provider (unit: years)
	*w*	The dynamic warning value of financial provider to network freight platform
**Threshold**	${L^{*}}_{i}(i=1,2)$	*i* = 1 The upper limit of credit line granted by financial provider to logistics providers/carriers, *i* = 2 The upper limit of credit line granted by financial provider to network freight platform

### 2.4 Model construction

#### 2.4.1 Taking the upstream and downstream enterprises of the platform as the main financing body

Logistics providers/carriers, as the main sources of financing, use the data provided by the network freight platform for credit financing. Financiers evaluate the transaction credit of a single financing enterprise, determine the upper limit of the credit line, and make the optimal credit line decision according to the maximum profit.

(1) Upper limit of the credit line

The financial provider changes the previous mode of evaluating credit only through financial statements, determines freight service income in combination with transaction credit, determines future discretionary cash flow according to freight service income, and then determines the upper limit of the credit line.

The expected freight volume of the financing enterprises in time *T* is set as *q*_1_, and *q*_1_ is a random variable. If the unit freight service price is *p* and the freight distance is *d*, the freight service income can be expressed as *pq*_i_*d*,and the corresponding freight service income based on the transaction credit *S* = *pq*_1_*dk*_1_. If the net profit rate of financing enterprise is *θ*_1_, the depreciation and amortization amount is *γ*_1_, the non-capitalized financial expense is *F*_11_, and the non-operating profit and loss is *F*_21_; thus, the operating net cash flow can be obtained as ${F^{*}}_{1}$

$${F^{*}}_{1}=S\theta_{1}+\gamma_{1}+F_{11}-F_{21}$$ (1)

If the initial monetary fund of the financing enterprise is *F*_01_, and the rigid expenditure such as bank loan and financial lease, is *e*_1_, then,

$$F_{1}={F^{*}}_{1}+F_{01}-e_{1}$$ (2)

Combining (1) and (2) is available

$$F_{1}=pq_{1}dk_{1}\theta_{1}+\gamma_{1}+F_{11}-F_{21}+F_{01}-e_{1}$$ (3)

The financial provider’s expectation of the future discretionary funds of the financing enterprise, that is, the upper limit of the credit line can be expressed as

$${L^{*}}_{1}=Epq_{1}dk_{1}\theta_{1}+\gamma_{1}+F_{11}-F_{21}+F_{01}-e_{1}$$ (4)

If the stochastic demand obeys the normal distribution, the freight volume generated by the demand also obeys the normal distribution $q_{1}∼N(\mu_{1},{\sigma^{2}}_{1})pq_{1}k_{1}\theta_{1}∼N(pk_{1}\theta_{1}\mu_{1},{\left(pk_{1}\theta_{1}\sigma_{1}\right)}^{2})$, then (4) can be expressed as

$${L^{*}}_{1}=pdk_{1}\theta_{1}\mu_{1}+\gamma_{1}+F_{11}-F_{21}+F_{01}-e_{1}$$ (5)

The upper limit of the credit line can be determined by the parameters in (5).

(2) Expected profit maximization

For the credit financing of highway transportation based on transaction credit, owing to the difficulty and expensive financing of small and medium-sized enterprises, the actual fuel and road and bridge fees needs to be paid in real time. Typically, logistics providers/carriers will accept the credit line of financial providers at a certain financing interest rate *r*_1_. Therefore, determining the credit line according to the expected profit maximization has become the key to credit financing.

We can also set the cost of credit financing for financial provider *c*_1_. To ensure that credit financing business is always conducted, there is always *c*_1_<*r*_1_. The repayment amount of financing enterprises is *x*_1_,and *x*_1_ is a non-negative random variable, its probability density function is *f*(*x*_1_), and its cumulative distribution function is *F*(*x*_1_), if *F*(*x*_1_) it is differentiable, strictly increasing, and continuous. ${\left(x_{1}\right)}^{+}=max(x_{1},0),\;{\left[L_{1}(1+r)-x_{1}\right]}^{+}$ is the maximum amount unpaid by the financing enterprise. To this end, the profits of financial provider *π* can be expressed as:

$$\underset{L_{i}}{\max}\;\pi (L_{1})=L_{1}(r_{1}-c_{1})-{\left[L_{1}(1+r_{1})-x_{1}\right]}^{+}$$

$$s.t.L_{1}\leq {L^{*}}_{1}$$

The expected profit is:

$$E\;\underset{L_{1}}{\max}\;\pi (L_{1})=L_{1}(r_{1}-c_{1})-\int_{0}^{L_{1}(1+r_{1})}F(x_{1})dx_{1}$$

$$s.t.L_{1}\leq {L^{*}}_{1}$$ (6)

Where $\int_{0}^{L_{1}(1+r_{1})}F(x_{1})dx_{1}=\int_{0}^{L_{1}(1+r_{1})}[L_{1}(1+r_{1})-x_{1}]f(x_{1})dx_{1}$ is the expectation of the unpaid amount of the financing enterprise, in Eq (6), *π*(*L*_1_) is the first derivative of *L*_1_,and we get $\frac{dE\pi (L_{1})}{dL_{1}}=r_{1}-c_{1}-\bar{F}[L_{1}(1+r_{1})]$, while the second derivative $\frac{d^{2}E\pi (L_{1})}{d{q_{1}}^{2}}=-f[L_{1}(1+r_{1})]<0$. Therefore, the profit function of the financial providers is a convex function about the credit line, and there is an optimal credit line. By making $\frac{dE\pi (L_{1})}{dL_{1}}=0$, you can get, $\bar{F}[L_{1}(1+r_{1})]=r_{1-}c_{1},\;\bar{F}(L_{1})=\frac{r_{1}-c_{1}}{1+r_{1}}$.

Therefore, in conclusion, the optimal credit line *L*_1_ decision of financial providers to logistics providers/carriers is determined in $\bar{F}(L_{1})=\frac{r_{1}-c_{1}}{1+r_{1}}{,L}_{1}\leq {L^{*}}_{1}$.

This shows that the optimal credit line of financial providers depends on the distribution of the repayment line of financing enterprises, credit interest rate of financial providers and the cost of credit financing under the condition of the upper limit of the credit line. Assuming that the repayment amount *x*_1_ obeys a uniform distribution, the constraint condition can be transformed into a uniform distribution interval $\left[0,{L^{*}}_{1}(1+r_{1})\right]$ and the corresponding density function $f(x_{1})=\frac{1}{{L^{*}}_{1}(1+r_{1})},\;\bar{F}(L_{1})=\frac{L_{1}}{{L^{*}}_{1}}=\frac{r_{1}-c_{1}}{1+r_{1}}$, and the credit line can be further obtained thus:

$$L_{1}=\frac{r_{1}-c_{1}}{1+r_{1}}(pdk_{1}\theta_{1}\mu_{1}+\gamma_{1}+F_{11}-F_{21}+F_{01}-e_{1})$$ (7)

The corresponding expected profit is:

$$\pi (L_{1})=L_{1}(r_{1}-c_{1})-\frac{L_{1}^{2}(1+r_{1})}{2L_{1}^{*}}$$ (8)

In Eq (7), because the highway transportation industry is completely competitive, the freight rate *p* is often determined by the market rather than by a single enterprise. Thus, it can be obtained through industry data. Net profit rate of financing enterprises *θ*_1_, depreciation and amortization amount of financing enterprises *γ*_1_, beginning monetary funds of financing enterprises *F*_01_, non-capitalized financial expenses of financing enterprises *F*_11_, non-operating profits and losses of financing enterprises *F*_21_, rigid expenditures, such as bank loans of financing enterprises, financial leases *e*_1_, and so on, can be determined via established data by checking bank accounts and final settlement returns. The credit financing cost of financial providers *c*_1_ also belongs to the industry’s deterministic value. Transaction credit *k*_1_, freight volume expectation *u*_1_ and credit interest rate *r*_1_ need to be judged in combination with the data provided by the network freight platform, and they become the key variables affecting the optimal credit line of financial providers.

**Proposition 1**: If other conditions remain unchanged, the credit line *L*_1_ and expected profit *π*(*L*_1_) increase with the transaction credit *k*_1_, expected freight volume *u*_1_ and credit interest rate *r*_1_.

Proof: When the credit interest rate *r*_1_ is increased by 0.1 unit, the added value of the corresponding credit line is $L_{1}^{\prime }-L_{1}=\frac{0.1+0.1c_{1}r_{1}}{\left(1+1.1r_{1})(1+r_{1}\right)}(pdk_{1}\theta_{1}\mu_{1}+\gamma_{1}+F_{11}-F_{21}+F_{01}-e_{1})>0$, and the corresponding expected profit added value is ${\pi (L_{1})}^{\prime }-\pi (L_{1})=\frac{0.21r_{1}^{2}-0.2r_{1}c_{1}+0.1r_{1}^{3}-0.1r_{1}c_{1}^{2}}{2(1+2r_{1})(1+r_{1})}(pdk_{1}\theta_{1}\mu_{1}+\gamma_{1}+F_{11}-F_{21}+F_{01}-e_{1})$. Based on *c*_1_<*r*_1_, there are $0.21r_{1}^{2}-0.2r_{1}c_{1}+0.1r_{1}^{3}-0.1r_{1}c_{1}^{2}>0.21r_{1}^{2}-0.2r_{1}^{2}+0.1r_{1}^{3}-{0.1r}_{1}^{3}>0$, namely *π*(*L*_1_)′−*π*(*L*_1_)>0. Therefore, the credit line and expected profit increase with the increase of credit interest rate, and the credit line and expected profit increase with the transaction credit *k*_1_ and freight volume expectation *u*_1_.

**Proposition 2:** If other conditions remain unchanged, the increase in credit line *L*_1_ brought about by the increase in credit interest rate *r*_1_ will increase more significantly than the increase in transaction credit *k*_1_, and the increase of the expected value of freight volume *u*_1_.

Proof: When the transaction credit *k*_1_ increases by 0.1 unit, the corresponding credit line added value is $L_{1}^{\prime \prime }-L_{1}=\frac{0.1{(r}_{1}-c_{1})pdk_{1}\theta_{1}\mu_{1}}{1+r_{1}}$. This combined with Proposition 1, proves that the difference between the credit line added value corresponding to the increase of credit interest rate and the credit line added value corresponding to the increase of transaction credit is $\frac{\left(0.1+0.21r_{1}c_{1}-0.1r_{1}+0.1c_{1}-0.11r_{1}^{2})pdk_{1}\theta_{1}\mu_{1}+(0.1+0.1r_{1}c_{1})(\gamma_{1}+F_{11}-F_{21}+F_{01}-e_{1}\right)}{\left(1+r_{1})(1+1.1r_{1}\right)}$, where $(1+r_{1})(1+1.1r_{1})>0,\;(0.1+0.1r_{1}c_{1})(\gamma_{1}+F_{11}-F_{21}+F_{01}-e_{1})>0,\;pdk_{1}\theta_{1}\mu_{1}>0$, and only the positive and negative signs $0.1+0.21r_{1}c_{1}-0.1r_{1}+0.1c_{1}-0.11r_{1}^{2}$ need to be determined for this purpose. According to the national standard interest rate control *r*_1≤_36%, this can be transformed into an extreme value problem with the following constraints:

$$y(r_{1})=0.1+0.21r_{1}c_{1}-0.1r_{1}+0.1c_{1}-0.11r_{1}^{2}$$

$$s.t.\;\left\{ \begin{array}{c} {0<r}_{1}\leq 0.36\\ {c_{1}<r}_{1} \end{array}\right.$$ (9)

By using the Lagrange multiplier method and introducing multipliers, it can be denoted as *α*,*β*

$$y^{\prime }(r_{1})=0.1+0.21r_{1}c_{1}-0.1r_{1}+0.1c_{1}-0.11r_{1}^{2}+\alpha (0.36-r_{1})+\beta (r_{1}-c_{1})$$ (10)

According to (10), calculate $\frac{\partial y^{\prime }(r_{1})}{\partial r_{1}}=0、\frac{\partial y^{\prime }(r_{1})}{\partial \alpha }=0、\frac{\partial y^{\prime }(r_{1})}{\partial \beta }=0$ and get the stagnation point *r*_1_ = *c*_1_ = 0.36, and the corresponding extreme value *y*(*r*_1_) = 0.11>0. From the function opening of *y*(*r*_1_) down to the decreasing function, we can see that *y*(*r*_1_) will increase with the decrease of *r*_1_, so we can get $\frac{\left(0.1+0.21r_{1}c_{1}-0.1r_{1}+0.1c_{1}-0.11r_{1}^{2})pdk_{1}\theta_{1}\mu_{1}+(0.1+0.1r_{1}c_{1})(\gamma_{1}+F_{11}-F_{21}+F_{01}-e_{1}\right)}{\left(1+r_{1})(1+1.1r_{1}\right)}>0$.

#### 2.4.2 Taking the platform as the main body of financing

As the main body of financing, a single logistics provider/carrier often has a small amount of financing; however, the evaluation process is consistent with the financing evaluation process when the amount is large. Because the network freight platform corresponds to *m*individual logistics providers/carriers, in practice, the financing needs of *m*individual logistics providers/carriers can be gathered, and the network freight platform can be used as the financing subject for credit financing, allocating the quota according to the repayment ability and business amount of each logistics provider/carrier.

(1) Credit decision

The service income of network freight platform comes from the difference between the contract amount collected from logistics providers and the contract finance paid to carriers. For *m*individual logistics providers, their freight service income can be expressed as $p\sum_{i=1}^{m}q_{i}\;d$, and the future discretionary funds of the network freight platform *F*_2_ can be obtained by referring to (3).

$$F_{2}=p\sum_{i=1}^{m}q_{i}dk_{2}\theta_{2}+\gamma_{2}+F_{12}-F_{22}+F_{02}-e_{2}$$ (11)

Referring to (5), the upper limit of the credit line between the financial providers and network freight platform can be obtained as follows:

$${L^{*}}_{2}=pdk_{2}\theta_{2}\sum_{i=1}^{m}\mu_{i}+\gamma_{2}+F_{12}-F_{22}+F_{02}-e_{2}$$ (12)

Referring to (6) the expected profit granted by financial providers to the network freight platform can be expressed as follows:

$$E\;\underset{L_{2}}{\max}\;\pi (L_{2})=L_{2}(r_{2}-c_{2})-\int_{0}^{L_{2}(1+r_{2})}F(x_{2})dx_{2}$$

$$s.t.L_{2}\leq {L^{*}}_{2}$$ (13)

Similarly, it can be obtained that the optimal credit line *L*_2_ decision of financial providers to network freight platform is determined in $\bar{F}(L_{2})=\frac{r_{2}-c_{2}}{1+r_{2}}{,L}_{2}\leq {L^{*}}_{2}$

Referring to Eq (7), the optimal credit decision of the financial providers for the network freight platform can be obtained as follows:

$$L_{2}=\frac{r_{2}-c_{2}}{1+r_{2}}(pdk_{2}\theta_{2}\sum_{i=1}^{m}\mu_{i}+\gamma_{2}+F_{12}-F_{22}+F_{02}-e_{2})$$ (14)

In Eq (14), the transaction credit *k*_2_, the expected value $\sum_{i=1}^{m}\mu_{i}$ of the freight volume of *m* individual logistics providers and the credit interest rate *r*_2_ need to be judged in combination with the data provided by the network freight platform, which are the key variables affecting the optimal credit line of financial providers. Because the expected value of freight volume $\sum_{i=1}^{m}\mu_{i}$ is a linear accumulation, the conclusions of Propositions 1 and 2 obtained by taking upstream and downstream enterprises of the network freight platform as financing subjects are still applicable to the mode of taking the platform as financing subjects.

(2) Warning value

Compared with a single logistics provider/carrier, the network freight platform gathers *m*individual logistics providers and carriers, bearing a huge highway transportation supply chain, and the credit line granted by financial providers is often larger than that of a single logistics provider/carrier. According to the assumptions put forward in the second part of this study, there is a linear correlation between the future business income of the network freight platform and the LPI and HFI. The following study will set dynamic early warning values in combination with LPI and the trend of HFI to hedge the risks brought about by transaction credit enhancement *m*.

For credit line *L*_2_ in credit period *T*, the monthly repayment line of the network freight platform is $\frac{L_{2}(1+r_{2})}{12T}$, and when the platform has repaid *t* months, the remaining repayment line is $L_{2}(1+r_{2})(1-\frac{t}{12T})$. According to the principle of financing self-compensation, the trend of the income capacity generated by future business operations of the platform is calculated, and the trends of net profit and repayment amount are compared to determine the dynamic early warning value.

The income generated from the operation of the network freight platform is mainly affected by the highway freight rate and logistics volume (transportation distance is a distance index and has stability), which correspond to the HFI and LPI, respectively.

It is assumed that the network freight platform’s revenue has the following linear relationship with the HFI and LPI:

$$S_{p}=\beta_{0}+\beta_{1}HFI+\beta_{2}LPI$$ (15)

**Fig 2:**
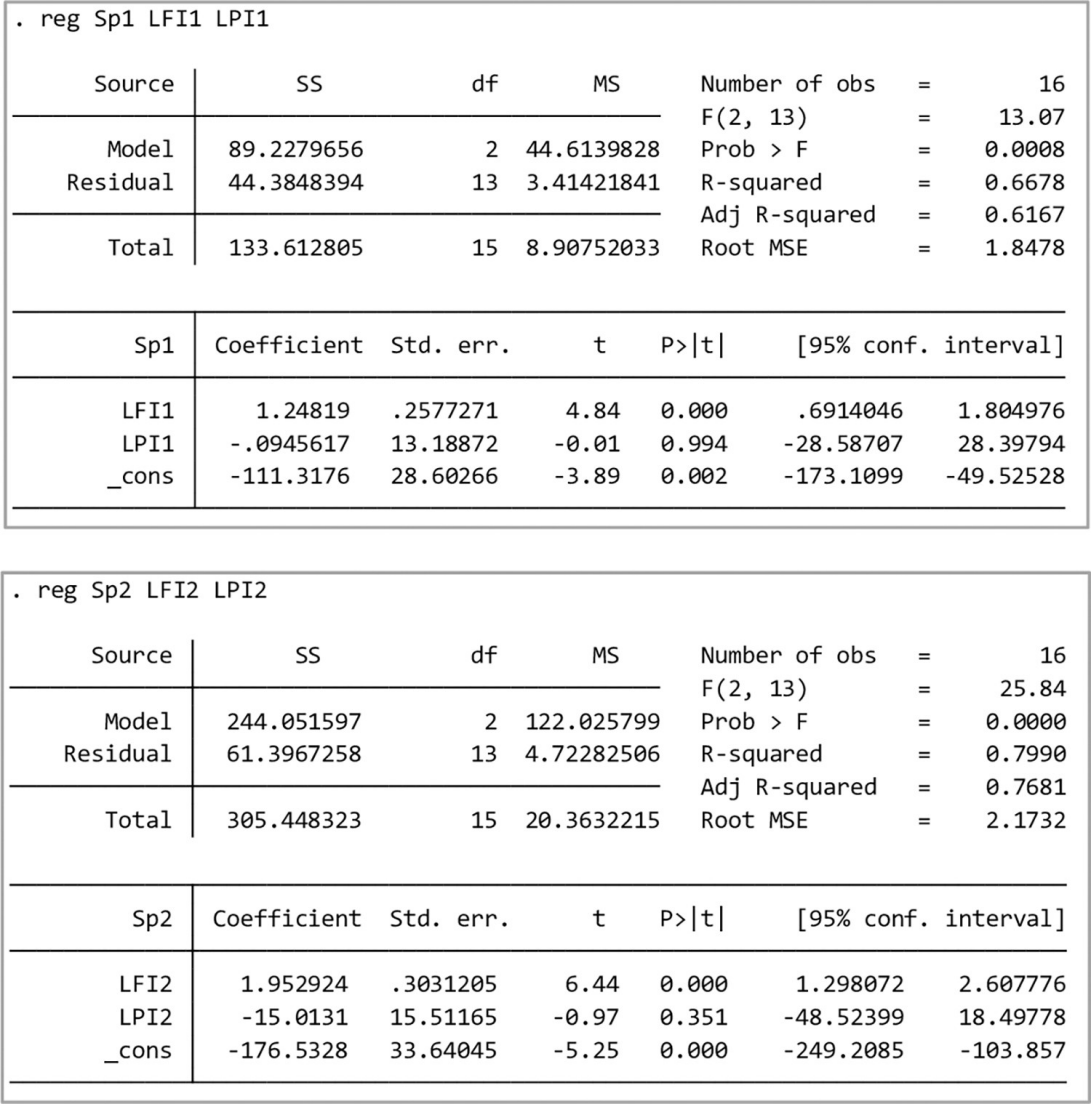
Regression output of sample network freight platform *S*_*p*_ and the HFI and LPI.

By selecting the revenues of LOGORY [23] and YMM listed network freight platform from 2019 to 2022 and the HFI and LPI in the corresponding time periods, the linear regression analysis results are illustrated in Fig 2 below.

There is a positive and significant correlation between platform revenue and the HFI, but no significant relationship exists with the LPI. The regression model *R*^2^ values are all above 0.6, and the regression model fits well. Considering that the two online network freight platforms, LOGORY and YMM, have a short time to market and only have annual data, and the amount of sample data may affect the significance of the model. The quarterly data from 2018 to 2022 of Debon Co., Ltd., a typical highway transportation enterprise, are selected as inspection samples, and the regression analysis results are obtained.

**Fig 3:**
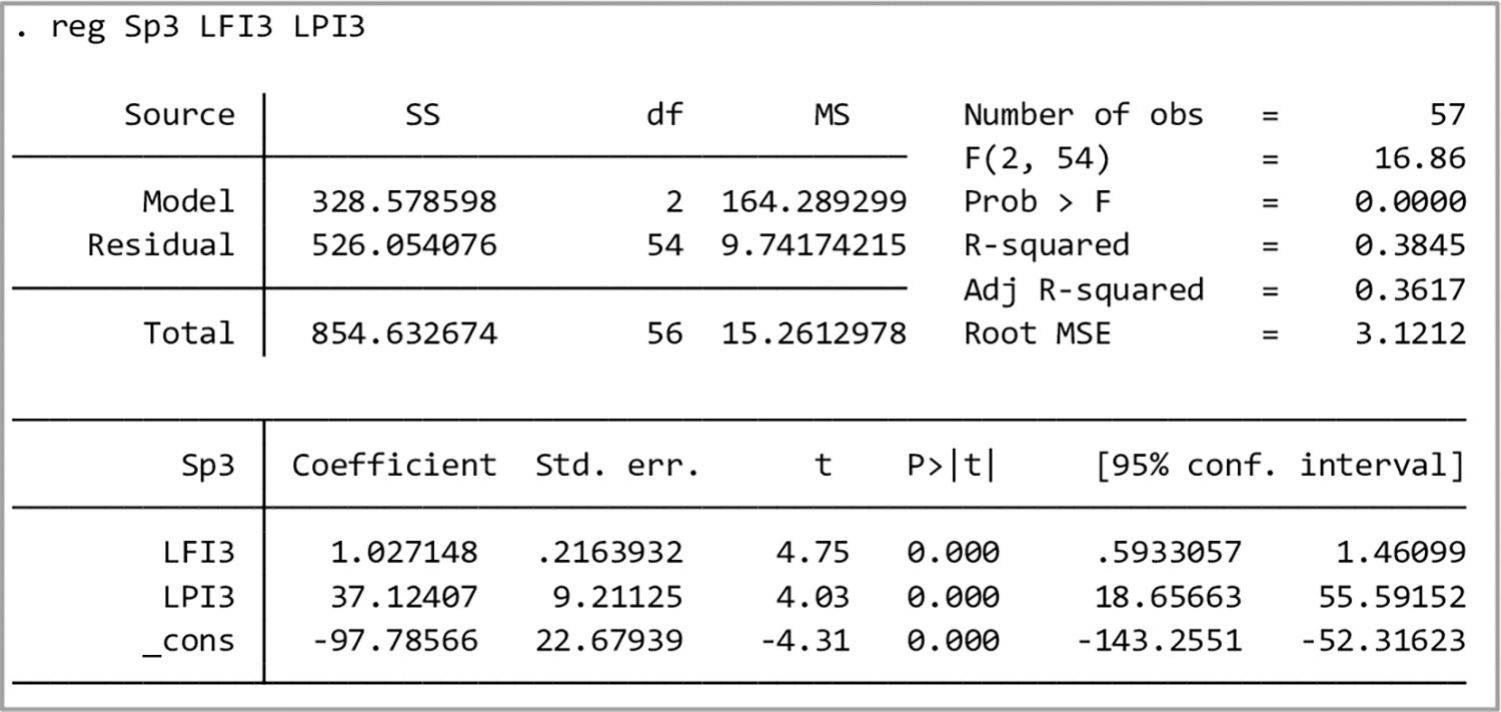
Regression output of test samples *S*_*p*_ with the HFI and LPI.

When the sample size increases, the platform revenue has a positive correlation with the HFI and LPI; the value *R*^2^ is close to 0.4, and the model fitting degree is also good (as shown in Fig 3). For this reason, the correlation coefficient *β*_0_,*β*_1_,*β*_2_ between platform revenue and the HFI and LPI can be determined from Eq (15), and the trend expression of predicting the future net profit of the network freight platform according to the HFI and LPI is given as follows.


$$I_{p}=(\beta_{0}+\beta_{1}HFI+\beta_{2}LPI)\theta_{2}k_{2}$$ (16)

The China Federation of Logistics & Purchasing publishes the HFI and LPI every month, while the accounting period of the logistics industry is usually three months or more. Therefore, according to the HFI and LPI data of the *t* period, the trend of net profit to be obtained in the platform *t*+3 period can be predicted, and so on, which can play an early warning role in the repayment ability of the network freight platform. When $I_{p}(1-\frac{t}{12T})>L_{2}(1+r_{2})(1-\frac{t}{12T})$, immediately *I*_*p*_≥*L*_2_(1+*r*_2_), the net profit of the network freight platform has repayment ability, and the dynamic early warning value is the unpaid amount, that is, $L_{2}(1+r_{2})(1-\frac{t}{12T})$. When *I*_*p*_≥*L*_2_(1+*r*_2_), the network freight platform’s repayment ability will have an impact, and the dynamic early warning value at this time is the repayment amount of each period, that is, $\frac{L_{2}(1+r_{2})}{12T}$, until the value *I*_*p*_ changes. Therefore, the dynamic early warning value of financial providers to the network freight platform can be expressed as

$$w=\left\{ \begin{array}{l} L_{2}(1+r_{2})(1-\frac{t}{12T}),\;I_{p}\geq L_{2}(1+r_{2})\\ \frac{L_{2}(1+r_{2})}{12T},\;I_{p}<L_{2}(1+r_{2}) \end{array}\right.$$ (17)

## 3. Results and discussion

Next, this study will use numerical examples to explore the influence of important parameters on credit lines and expected profits of financial providers based on different models. We maintain the following assumptions in the whole model: *c*_1_ = *c*_2_ = 2.6% (according to Choice2022 ranking of financing costs of financial institutions), *p* = 0.31,*d* = 1215 (calculated according to the data of China Highway Logistics Freight Rate Weekly Index Report from March 7, 2022, to March 11, 2023, released by China Federation of Logistics & Purchasing) [24]. Notably, *θ*_1_ = 4.4% (According to several business statistics, the average net interest rate of 43 logistics listed companies), *θ*_2_ = 2% (Robinson Global Logistics has a net interest rate of 3.8% in 2022 and LOGORY 2022Q3 has a net interest rate of 0.1%). $\gamma_{1}=29,310,025,\;F_{01}=72,559,770,\;F_{11}=2,804,801,\;F_{21}=0,\;e_{1}=76,455,692$ (obtained according to company code 603813, 2021 annual report). $\gamma_{2}=10,341,000,\;F_{02}=728,838,000,\;F_{12}=4,925,000,\;F_{22}=0{,\;e}_{2}=51,100,000$ (obtained according to the company code 02482. HK, 2021 Annual Report),*T* = 1.

### 3.1 Impact of transaction credit

Based on the above-stated settings, we first consider the impact of transaction credit on the credit line and expected profit. We keep the credit interest rate and expectation freight volume unchanged, that is, $r_{1}=r_{2}=10\%\mu_{1}=1,200,000\sum_{i=1}^{m}\mu_{i}=16,000,000$. We calculate the impact of the change in transaction credit on the credit line and expected profit of financial providers under different credit models.

**Fig 4:**
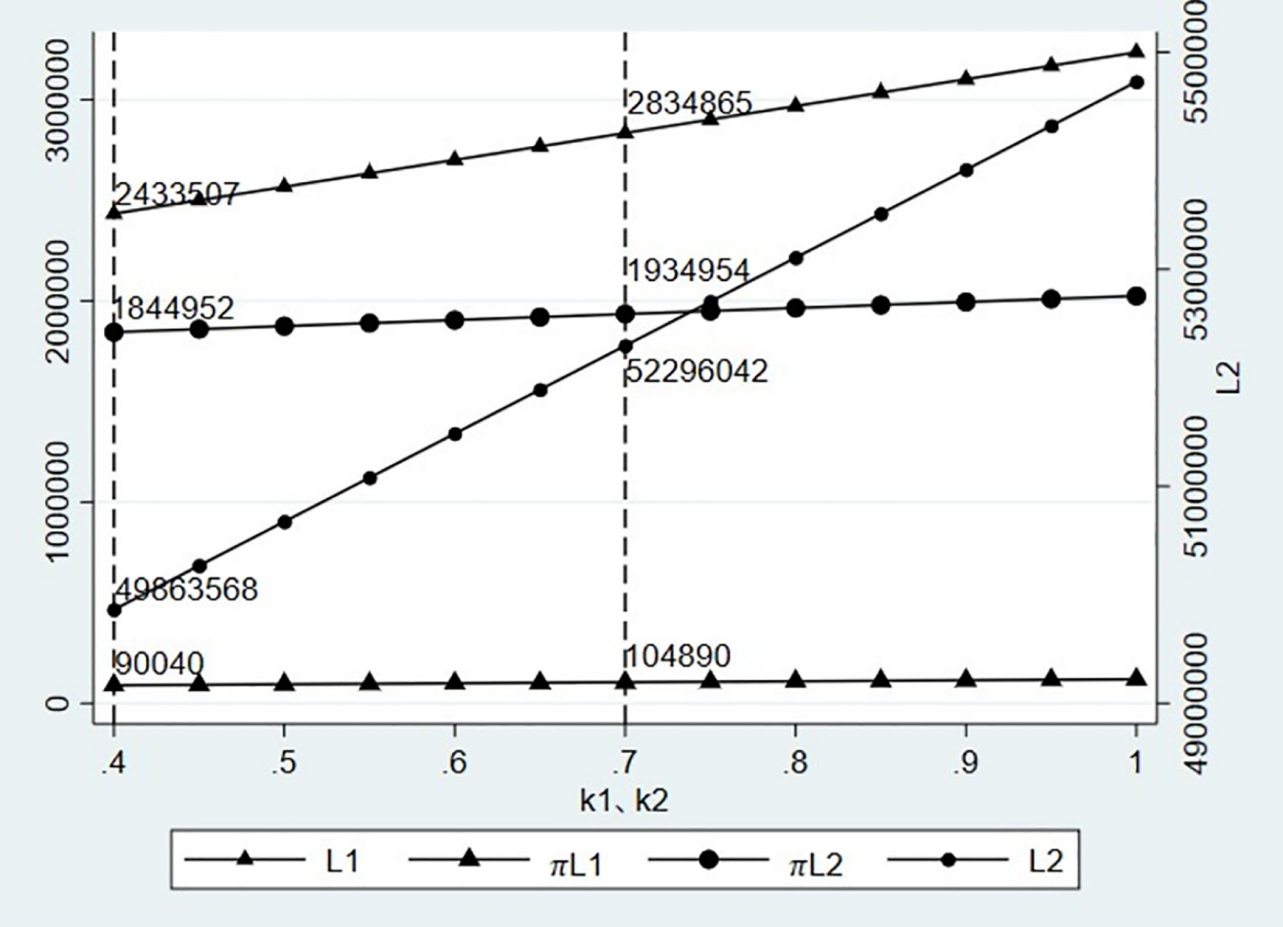
Impact of transaction credit change on credit line and expected profit.

**Fig 5:**
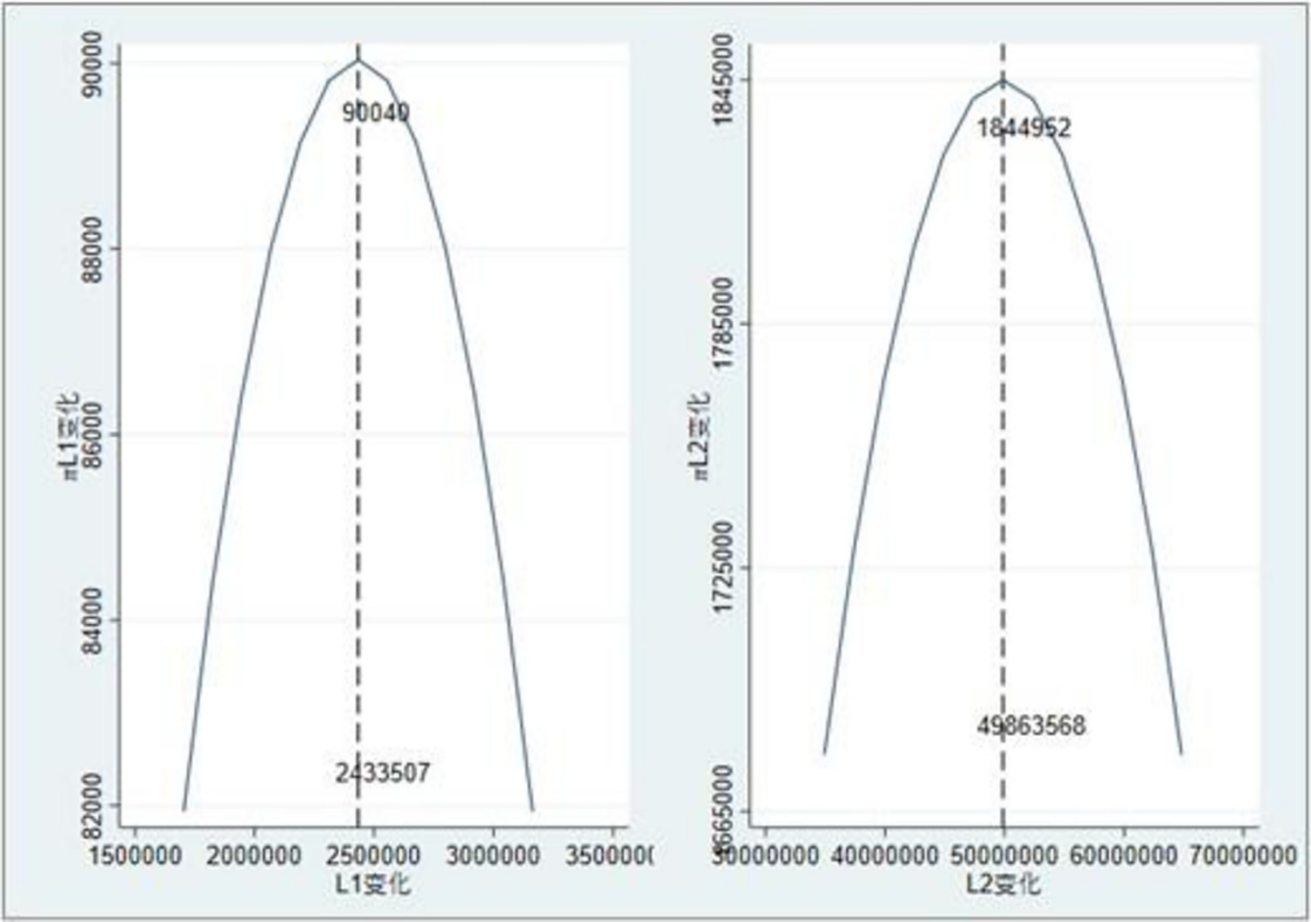
Changes in expected profits under different credit lines.

According to Fig 4, with the gradual increase in transaction credit, the credit line and expected profit of the model with the logistics provider/carrier as the credit subject and the network freight platform as the credit subject increase with the transaction credit, which is consistent with the conclusion of Proposition 1. Simultaneously, when transaction credit *k*_1_ = *k*_2_ = 0.4, regardless of the mode, the financial providers’ expected profit will not increase with the credit line (as shown in Fig 5). The credit line has optimal values of *L*_1_ = 2,433,507,*L*_2_ = 49,863,568, and it is consistent with the corresponding credit line in Fig 4, which proves the effectiveness of the model.

### 3.2 Impact of freight volume

We keep the transaction credit and credit interest rate unchanged *r*_1_ = *r*_2_ = 10%,*k*_1_ = *k*_2_ = 0.7 and calculate the impact of the change in the expected freight volume under different modes on the credit line and expected profit of financial providers.

**Fig 6:**
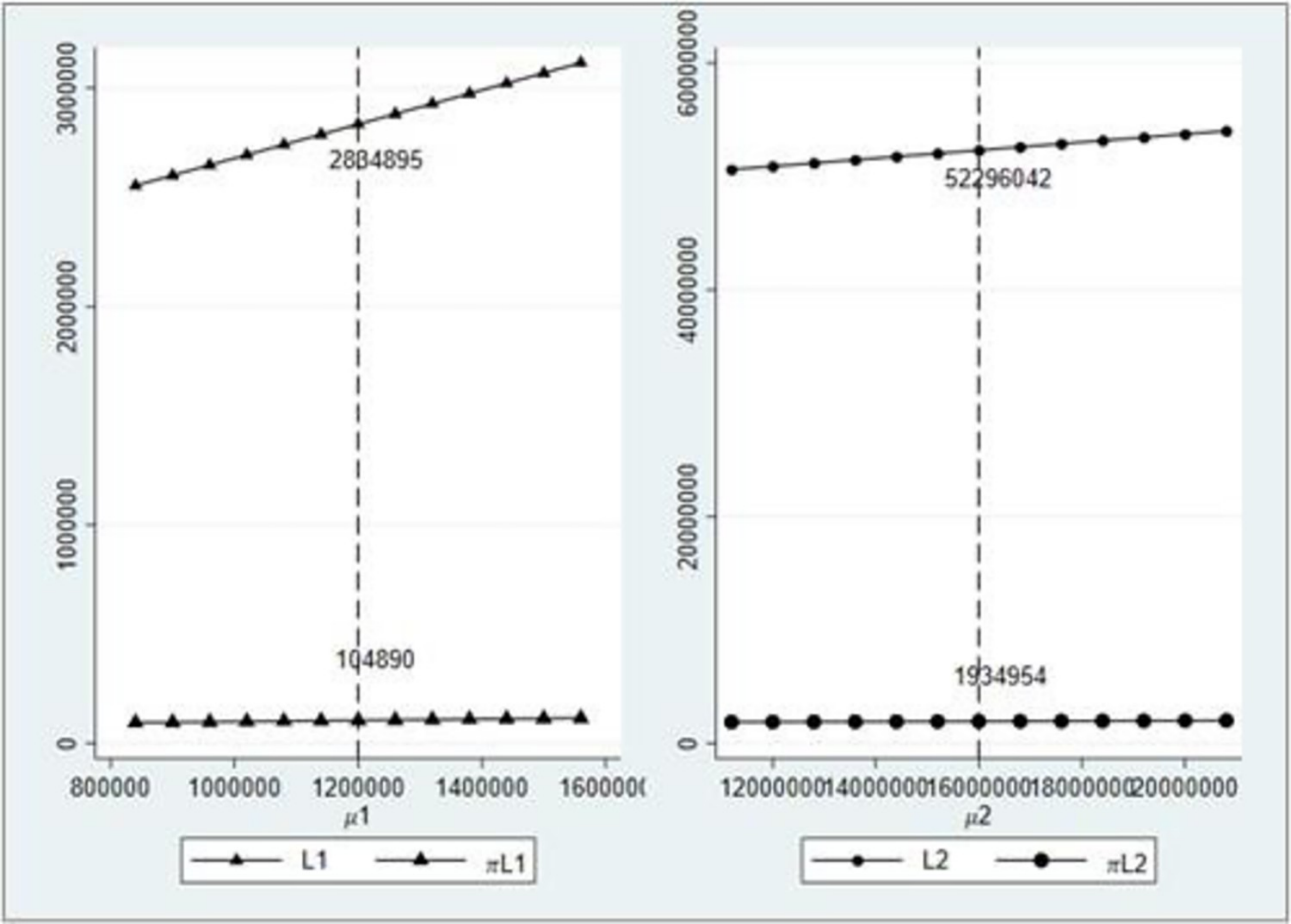
Influence of expected value change of freight volume on credit line and expected profit.

As Fig 6, with an increase in the expected freight volume, the credit line and expected profit of financial providers under different credit modes also show an increasing trend, and the growth rate of the credit line is evident compared with the growth rate of the expected freight volume. Also, the credit line and expected profit under the corresponding freight volume are consistent with those in Fig 4, that is, when *μ*_1_ = 1,200,000, there is *L*_1_ = 2,884,895,*πL*1 = 104,890, and when *μ*_2_ = 16,000,000,there is *L*_2_ = 52,296,042,*πL*2 = 1,934,954.

### 3.3 Impact of credit interest rate

We keep the transaction credit and expected freight volume unchanged, that is, $k_{1}=k_{2}=0.7\mu_{1}=1,200,000\sum_{i=1}^{m}\mu_{i}=16,000,000$, and calculate the impact of the change of credit interest rate on the credit line and expected profit of financial providers under different modes.

**Fig 7:**
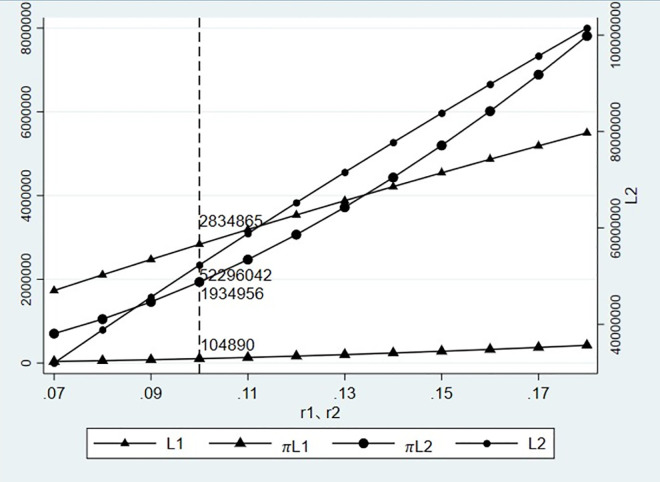
Influence of credit interest rate change on credit line and expected profit.

Fig 7 shows that with an increase in the credit interest rate, the credit line and expected profit that financial providers are willing to increase under different modes, and the increase is more significant than that caused by transaction credit and expected freight volume, which is consistent with the conclusion of Proposition 2. This is consistent with the principle of high risk and high yield in actual operations. Under higher interest rates, financial providers are willing to bear certain risks and provide more credit lines to financing enterprises. The credit line and expected profit under the same interest rate are consistent with those in Figs 4–6.

### 3.4 Two-dimensional credit matrix

For specific financing subjects, the expected value of freight volume is usually measured directly, while the evaluation of transaction credit and credit interest rate, especially transaction credit, is complicated and uncertain. Therefore, for specific financing enterprises, we can describe the two-dimensional credit matrix of the financing subject according to different transaction credit and credit interest rates and provide financial providers with an intuitive credit reference.

Referring to the customer rating operation in practice, we divide the transaction credit into different credit ratings according to different credit values. Transaction credit is classified as C and CC by 0.4 ~0.55, CCC-BB by 0.6 ~ 0.7, BBB-A + by0.75 ~ 0.85 and AA-AAA by 0.9 ~1. Taking logistics providers/carriers as an example, the corresponding two-dimensional credit matrix can be obtained, as shown in Table 2 (*μ*_1_ = 12,00,000).

**Table 2:** Two-dimensional credit matrix of logistics provider/carrier.

transaction credit / credit interest rate	C、CC	CCC-BB	BBB-A+	AA-AAA
	0.4~0.55	0.6~0.7	0.75~0.85	0.9~1
**7%**	37665286 1548853	41145532 1691966	44128600 1814634	47111668 1937302
**8%**	37665286 1883264	41145532 2057277	44128600 2206430	47111668 2355583
**9%**	37665286 2211540	41145532 2415884	44128600 2591037	47111668 2766190
**10%**	37665286 2533847	41145532 2767972	44128600 2968651	47111668 3169330
**11%**	37665286 2850346	41145532 3113716	44128600 3339462	47111668 3565207
**12%**	37665286 3161194	41145532 3453286	44128600 3703650	47111668 3954015
**13%**	37665286 3466540	41145532 3786845	44128600 4061393	47111668 4335941
**14%**	37665286 3766529	41145532 4114553	44128600 4412860	47111668 4711167
**15%**	37665286 4061300	41145532 4436562	44128600 4758214	47111668 5079867
**16%**	37665286 4350990	41145532 4753018	44128600 5097614	47111668 5442210
**17%**	37665286 4635728	41145532 5064065	44128600 5431212	47111668 5798359
**18%**	37665286 4915639	41145532 5369841	44128600 5759156	47111668 6148472

The credit elements in each two-dimensional cell are the upper limit of the credit line and the optimal credit line, from top to bottom. Among them, the upper limit of the credit line is related to the transaction credit, while the optimal credit line is related to the transaction credit and credit rate. For example, according to the two-dimensional credit matrix in Table 2, taking the expected freight volume of logistics providers/carriers of financing enterprises as an example, when the transaction credit evaluation value of financial providers for financing enterprises is 0.4~0.55, if the credit rate granted to financing enterprises is 7%, then the upper limit of the credit line is 37,665,286. The optimal credit line is 1,548,853. If the credit rate is 10%, the optimal credit line is 2,533,847. When the transaction credit evaluation value of the financial providers for the financing enterprise is 0.6~0.7, if the credit rate given to the financing enterprise is 7%, the upper limit of the credit line is 41,145,532, and the optimal credit line is 1,691,966. The practical significance of the forementioned is that in the actual business operation process of financial providers, this two-dimensional matrix can enable not only project managers to provide a reference for the pre-credit line when negotiating with financing enterprises but also risk control personnel to have a reference when determining the credit line. The two-dimensional credit matrix of the network freight platform can also be calculated using the same method, which is not repeated here.

## 4. Conclusions

This study takes the platform-type highway transportation supply chain as the object. First, by expounding the platform-type highway transportation supply chain, two credit financing modes are defined: logistics provider/carrier and the network freight platform as the main body. Second, based on the principle of “true business and closed-loop transaction,” the transaction credit is determined through the credit system of the entire chain, and the upper limit of the credit line is determined according to the self-compensation of financing from future discretionary funds. Third, a model of expected profit maximization is constructed, and the optimal credit line under the two modes and the dynamic early warning value under the network freight platform mode as a financing subject is determined. Finally, using numerical examples, this study analyzes the influence of three factors, namely, transaction credit, freight volume and credit interest rate on credit line and expected profit.

The main conclusions of this study are as follows. Notably, first, this study establishes profit maximization models with upstream and downstream enterprises as well as platforms as financing subjects and provides the expression of the optimal credit line. Second, under the guidance of data reaching and price marking, combined with the HFI and LPI, the dynamic early warning value under the network freight platform mode as the main financing body is set. Third, it analyzes the influence of transaction credit, freight volume and credit interest rate on credit line and expected profit under different credit modes. Both credit line and expected profit increase with the transaction credit, expected freight volume and credit interest rate, and the increase brought about by the credit interest rate is more significant than that caused by transaction credit and freight volume. Fourth, the two-dimensional credit matrix of the financing subject is described by transaction credit and credit interest rate, which provides financial providers with an intuitive credit reference.

Based on the research conclusions, this study puts forward the following management suggestions: First, as the main body of financial providers to build transaction credit, the network freight platform should strengthen its own digital construction and enhance the recognition of financial providers for the platform and upstream and downstream financing enterprises. The network freight platform should strengthen its own digital construction from the aspects of information investment, platform data intelligent collection, platform data matching degree and verifiability, system stability, data capacity, data security, and so on, to enhance transaction credit; break the information barriers between banks and enterprises; improve the willingness of financial providers to grant credit; and increase the financing availability of small, medium and micro transportation logistics enterprises. Second, logistics providers, carriers, and other small and medium-sized transportation logistics enterprises, in the case of their own limited digital level, should strengthen cooperation with the network freight platform. With the digital ability of the network freight platform for contracts, invoices, waybills, receipts, statements, and so on, the authenticity and credibility of business income are increased. Third, financial providers, as an important starting point for enhancing the vitality of market players, should actively innovate the credit model for small and medium-sized logistics enterprises. The traditional credit granting model of financial providers mainly focuses on the pledge and mortgage of goods, and 95% of China’s huge number of vehicles are owned by individuals, and these vehicles are operated through affiliations, and do not belong to the assets of small and medium-sized logistics enterprises. Financial providers need to change the credit granting mode; take business as the basis of credit granting; increase the credit of small, medium, and micro transportation logistics enterprises through the data of small, medium, and micro transportation logistics enterprises gathered by the network freight platform; use the transaction credit formed by data to increase the credit of small, medium and micro transportation capacity logistics enterprises; and use the credit granting exposure of financial institutions covered by the self- compensation of business income as the judgment of the credit line. These can help to not only solve the practical problems of small and medium-sized logistics enterprises but also promote the development of inclusive finance.

Subsequent this study can be extended from the following directions. First, this study considers the profit maximization of financial firms at present, and it can also consider the profit maximization of financing enterprises in the future, to seek a balance in mutual profit maximization. Second, this study sets transaction credit as a certain value, but the evaluation of the specific transaction credit and what evaluation indicators ought to be established can be further considered in the future studies. Third, there are many participating enterprises in the platform-based highway transport capacity supply chain. Whether shippers choose the logistics services of logistics providers or provide logistics services through the network freight platform, and game between logistics providers and platforms can become the content of subsequent expansion.

## Supporting information

S1 Data (XLSX)

S1 File (PDF)

S2 File (PDF)
