# Supplementary material for: Granting analysis of the credit financing of the platform-type highway transportation supply chain
Source: PLoS One. 2024 Jul 10;19(7):e0303807. doi: 10.1371/journal.pone.0303807 (PMC11236115; doi:10.1371/journal.pone.0303807)
Supplement: S1 File — (PDF) [file pone.0303807.s002.pdf]

基于百度指数的公路运价指数 RO-ELM 预测

朱 曦[1]，赖应良[1]，段雨彤[2]

(1. 昆明理工大学 建筑工程学院，昆明 650093；2. 云南大学 历史与档案学院，昆明 650093)

摘要：公路运价指数是公路运输市场波动的衡量指标，对中国的公路运输业有重要的预示功能。利用极限学习机(ELM)的神经网络模型快速、低成本预测公路运价指数。以各百度指数与公路运价指数的相关性确定各分量对公路运价指数的影响，进而利用 ADF 平稳性检验与 Johansen 协整检验构建输入序列，最后运用时域优化思想优化输入变量，在 ELM 神经网络模型内输出预测值。结果表明：基于滚动窗口的 ELM 模型的 MAPE 与 RMSE 分别为 1.85% 与 25.17，比单一 ELM 模型在平均绝对百分比误差和均方根误差上都有提升，预测结果与指数波动相符，可以为公路运价指数的走向提供决策参考。

关键词：公路运输经济；公路运价指数；极限学习机；预测

中图分类号：F542 **文献标志码**：A **文章编号**：1671－1807(2021)01－0179－06

截止 2019 年，中国公路货运量突破 416 亿吨，货物周转量接近 8 万亿吨公里，载货汽车保有量超过 1 300 万辆，已发展成为全球第一大市场[1]。但中国公路货运领域，小、散、乱、弱的局面长期存在，公路货运信息不对称、公路运价体系不透明，这些弊端严重制约了公路货运向前迈进的步伐。自 2015 年以来，国务院批准设立的中国唯一物流与采购行业综合性社团中国物流与采购联合会公开发布公路运价指数。目前，采集的价格数据涵盖了全国 9 大物流区域、38 个重点城市、74 个物流节点平台、1 406条公路运输线路，200 万辆货运车辆，数据采集面广、量大，显著提高了数据的全面性、代表性，得到专家甚至社会的广泛认可[2]。故选择公路运价指数作为研究对象。

关于公路运价指数的研究，主要集中于定性解释，公路运价指数的预测相对较少。中国学者顾敬岩等发现公路货运市场价格体系扭曲，价格调节机制失灵，分析其内在机理后提出多角度政策建议[3]。戴宏提出了中国公路货运物流服务价格存在的主要问题，从融资渠道、创新模式方面给出针对性建议。公路运价波动内在影响因素众多，定性研究指出了价格波动的内在原因，缺乏波动趋势的解释性验证。伴随人工智能技术的进步，已经有学者在公路运输行业使用数字化工具进行分析预测[4]。胡欣基于公路物流车辆的 GPS 数据，构建了公路物流指数模型，并利用 RBF 神经网络来预测公路运价指数[5]。彭建良等利用了记忆神经网络混合时间序列自回归来对指数进行预测[6]。

公路运价指数反映的是中国公路运输市场的商品及服务的价格水平，其波动受到运输市场供需、宏观政策及经济形势等影响。随着互联网的兴起，人们获取信息的方式正在由传统渠道向网络渠道转移[7]。生产者与消费者在互联网上留下搜索记录，网络也记录了生产者与消费者的浏览痕迹，这些痕迹反映了他们本身关注点，显示了他们日常生产中的生产、消费的趋势。这种趋势本身折射出社会经济的冷热，在商品经济中体现为价格与交易量的变化，该相互依存的关系可以作为一种信息资源来预测公路运输市场的冷热[8]。利用网络搜索指数对经济活动的监测及预测研究在外国已经开展了大量研究，这些研究多出现于 2008 年以后，还尚未形成系统的研究体系，基于 Google 指数的流感预测[9]、失业率预测、汽车及房地产[10]等行业销售量预测都取得了较高的准确度，而且网络数据的即时性能够有效地弥补传统的监测方法的不及时，具有更强的时效性。ELM 模型是一种便捷快速的神经网络模型，它规避了陷入局部最优的问题，而且极大地提高了神经网络的学习速度和推广性能，更加

收稿日期：2020－09－04

基金项目：云南省哲学社会科学规划项目(YB201875)。

作者简介：赖应良(1963—)，男，云南昭通人，昆明理工大学建筑工程学院，副教授，硕士生导师，高级工程师，研究方向：质量管理工程；朱曦(1995—)，云南昆明人，昆明理工大学建筑工程学院，硕士生，研究方向：公路经济。

(C)1994-2023 China Academic Journal Electronic Publishing House. All rights reserved. http://www.cnki.net

适合实际应用。目前已经在天气预测和农业信息技术识别中得到了运用[11]。

现在已有的基础上,提出一种基于大数据ELM建模的方法,利用网民使用率为84.6%的百度搜索引擎作为信息入口,对百度指数进行时域优化。同时发挥人工智能算法的优势快速预测运价指数变化。依靠互联网信息的高容量与便捷性,可以研究消费者的网络搜索行为,从而相对传统数据采集计算全周期内提前预测指数信息[12-13]。

1 基本原理

1.1 ELM极限学习机模型

Huang等提出的极限学习机(extreme learning machine,ELM)是一种单隐层的前馈神经网络,其模型以其快速的学习速度和泛化能力被广泛应用于许多领域,该模型的重点是随机生成输入权值和偏差,不需要对隐含层参数进行调整,可通过简单的矩阵计算得到输出权值,计算时间短[14-16]。

Huang等发现,在前馈神经网络中,当隐含层神经元的激励函数$G(x)$无限可微时,对于任意的N个不相同的样本(x_i, y_i),其中,$x_i=[x_{i1}, x_{i2}, \cdots, x_{in}]^{\mathrm{T}} \in R_n$, $y_i=[y_{i1}, y_{i2}, \cdots, y_{ik}]^{\mathrm{T}} \in R_k$,则一个具有l个隐层节点,激励函数为

$$\boldsymbol{y}_i = f(\boldsymbol{x}_i) = \sum_{j=1}^{l} \boldsymbol{\beta}_j G(\boldsymbol{w}_i \cdot \boldsymbol{x}_i + \boldsymbol{b}_i),\ \boldsymbol{w}_i \in R^n,\ \boldsymbol{\beta}_j \in R^k \tag{1}$$

其中,$i=1,2\cdots,N$,$j=1,2,\cdots,l$;$\boldsymbol{w}_j=[w_{i1}, w_{i2}, \cdots, w_{in}]^{\mathrm{T}}$是连接输入层到第j个隐层节点的输入权重;$\boldsymbol{\beta}_j=[\beta_{j1}, \beta_{j2}, \cdots, \beta_{jk}]^{\mathrm{T}}$是连接第$j$个隐层节点到输出节点到输出节点的输出权重;$b_j$是第$j$个隐层节点的偏差值;$\boldsymbol{w}_j \cdot \boldsymbol{x}_j$表示向量$\boldsymbol{w}_j$和$\boldsymbol{x}_i$的内积。激励函数$G(x)$可以选择为"Sigmoid"、"Tansig"、"Sine"或"RBF"等。若隐含层神经元个数K小于等于训练集样本数N,则对任意的输入层神经元与隐含层神经元的连接权值w和隐含层神经元的偏置b,前馈神经网络的训练误差可以逼近一个任意小的数$\varepsilon(\varepsilon>0)$。其中,$w$和$b$只需要在前馈神经网络训练前随机选取,并在训练过程中可保持不变。而隐含层与输出层之间的连接权值β可通过最小二乘法求解以下问题获得。

$$\min_{\beta} = \|\boldsymbol{H}\beta - \boldsymbol{T}\| \tag{2}$$

其中,$\boldsymbol{H}$为隐含层输出矩阵;$\boldsymbol{T}$为前馈神经网络的输出矩阵。可解得

$$\beta = \boldsymbol{H}^{+}\boldsymbol{T} \tag{3}$$

其中,$\boldsymbol{H}^{+}$为矩阵$\boldsymbol{H}$的Moore-Penrose广义逆矩阵。且可证明求得的解$\boldsymbol{\beta}$的范数最小且唯一。

1.2 滚动时域优化

由于公路运价指数以周为频率统计,同时节假日不予发布,而百度指数以天为频率生成,故整个时间跨度内百度指数与公路运价指数的结构关系难以准确反映。利用时域优化思想可以削弱公路运价指数的遗漏对样本的干扰,较准确反映百度指数与公路运价指数间的复杂关系。在时域优化滚动机制中,间隔周期选择过大则求解问题的规模增大同时无法快速响应系统中发生的干扰,过小则调度过于频繁影响了系统的稳定性。滚动策略应选取符合公路运价指数波动的滚动机制来充分反映百度指数对运价指数的而影响[15]。

滚动时域优化是预测控制中滚动优化原理的一种具体表现形式。滚动时域优化与传统控制优化不同之处在于它并非全局优化,也不是一次性优化,而是一种有限范围内的连续滚动优化。如图1所示,采用基于时间的窗口(rolling windows)应首先定义完工窗口,从时间当前时刻t_0开始,以固定窗口步距T_{p}为滚动窗口时间步长,将所有完工窗口内任务移除,再把等待窗口内任务移入预测窗口$W(T)$,逐次以时间间隔t向后移动并重复上述步骤,最终得到滚动优化的时间序列。

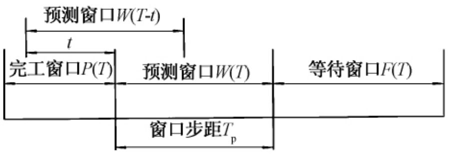

图1 滚动窗口示意图

2 实验与分析

2.1 数据集

为了验证ELM模型的有效性,在Inter(R)Core(TM)i5-9400F CPU@2.90GHz处理器、8GB内存的台式机上进行实验,使用Python程序设计语言对数据集进行处理。数据源为2016年9月至2019年11月公路物流运价周指数数据和同期内所有日的百度指数作为研究对象。百度指数是百度公司发布的开源的数据资源,它记录了自2011年以来的给定关键词的当日搜索量。公路运价指数如图2所示。

2.2 搜索关键词选择与经济内涵分析

清华大学的张锡平等[16]经25个月调研涵盖了中国6大地区共计14个省市,获得的2003年中国汽车运输成本结构。其中燃油费占25.6%,固定税

(C)1994-2023 China Academic Journal Electronic Publishing House. All rights reserved. http://www.cnki.net

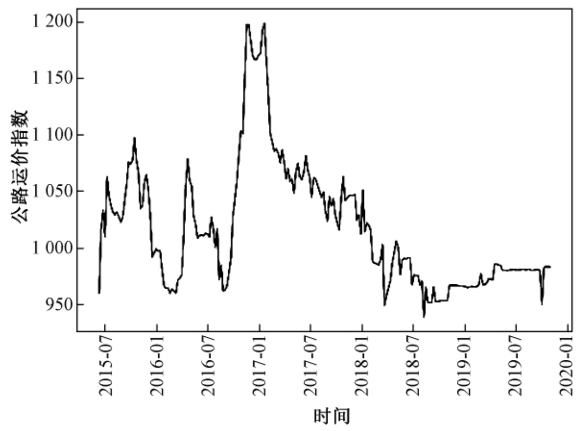

图 2　公路运价指数

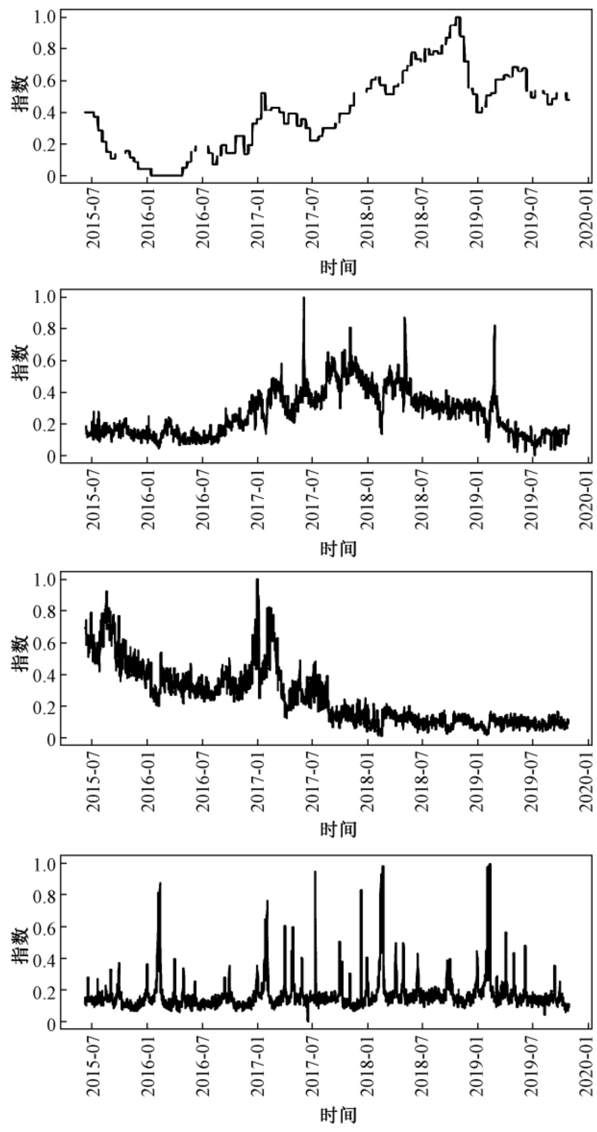

图 3　各关键词百度指数

费所占比重为 17.3%，人工成本占 17.0%，折旧成本所占比重 9.5%。过路过桥费与罚款所占比重合计为中国汽车运输成本的 12.8%，对于主要从事长途运输的重型和中型货车，过路过桥费和罚款所占比重更高，分别达到 21.0%和 18.8%。

基于此根据公路运价的影响因素构成初选关键词：选取燃油费用、道路通行费、人工费用和维护修理费四大类运价构成因素。然后根据百度指数的关键词推荐功能，逐一输入关键词，得到 30 多个"报废汽车"、"oil0"、"oil95"、"oil98"、"工资"、"货车价格"、"汽车保险"、"CPI"、"柴油价格"、"油价"、"油价调整"、"二手货车"、"过路费"等反应公路运输市场的关键词。此处也爬取了公路运输的油价数据选取关键词如图 3 所示。

最后根据皮尔逊相关系数，选取相关性最佳的关键词组成预测运费基础的数据，进以进行检测。如表 1 所示在燃油费上"oil0"相比"oil98"的百度指数时间序列具有 0.33 的较佳相关系数，故选取该数据作为燃油费用的代表建模。

根据关键词的统计结果与可知，"报废汽车"、"二手货车"与"过路费"的时间序列均呈现出不同的波动特性，其内在经济意义不同：

表 1　关键词的统计特征

序列	均值	标准差	偏度	峰度	相关系数
二手货车	222.8	123.4	1.06	0.67	0.28
报废汽车	336.6	127.2	0.73	0.16	−0.34
过路费	268.7	107.0	4.18	22.08	−0.45
oil0	6251.2	757.8	0.16	−0.74	0.33
oil98	7917.7	221.7	0.40	−0.97	0.26
油价	7924.0	5146.3	2.94	10.76	−0.15
cpi	2352.4	1169.9	4.77	32.50	−0.01
工资	1015.4	237.7	0.29	0.55	−0.16

1)"过路费"的百度指数趋势具有周期性，与公路运价指数具有最大系数−0.45，是模型内对公路运价指数的最大影响因素。其波动趋势的周期性极值往往与节日事件相关。如 2016 年 2 月、2017 年 2 月、2018 年 2 月与 2019 年 2 月都出现了极大值，对应为春节高速公路过路费不予征收。类似的国庆长假也使得"过路费"的百度指数出现波峰。免过路费的重大事件与公路运价指数的下降呈显著影响，反映出节假日对公路运输市场的影响。

2)"报废汽车"的百度指数与有公路运价指数的相关系数为−0.34，在 2016 年后呈现缓慢震荡上升，其波峰一般出现在春秋两个季节，同时会受到不定期的额政策影响，如 2017 年颁布新的《机动车强制报废标准规定》使得"报废汽车"的百度指数大

(C)1994-2023 China Academic Journal Electronic Publishing House. All rights reserved.　http://www.cnki.net

幅增加。因此,“报废汽车”的百度指数反映了公路运输市场的政策性与季节性等。

3)“oil0”时间序列是0号柴油的市场波动真实反映,其变化随石油市场机构调整变化,波动性较小。同时与公路运价指数相关系数为0.33,反映出公路运输市场油价供需正向作用的规律。

4)“二手货车”的百度指数自2015年6月以后呈现震荡下降,与公路运价指数的相关性为0.28。显示出二手货车市场的繁荣与运输交易成本之间的关联性,可体现折旧费对影公路运输市场的影响。

2.3　ADF平稳性检验与协整验证

ADF(augmented dickey－fuller)平稳性检验能够确定时间序列变量的稳定性。对于p阶的VAR

$$\boldsymbol{y}_t = A_1\boldsymbol{y}_{t-1} + \cdots + A_p\boldsymbol{y}_{t-p} + B\boldsymbol{x}_t + \boldsymbol{\varepsilon}_t, t = 1, 2, \cdots, T \quad (4)$$

其中,$\boldsymbol{y}_t$是一个$(n\times 1)$向量,所含的n个经济变量皆为I(1)序列;$\boldsymbol{x}_t$是$\boldsymbol{y}_t$生成过程中可能包含的确定性成分(如截距,趋势项等),为d维向量;$\boldsymbol{\varepsilon}_t$是一个新息向量。若其所有特征根均小于1时,时间序列平稳;若上述p阶回归模型非平稳。ADF方法的零假设为$H_0:\rho=0$。若拒绝该零假设,则时间序列平稳,否则时间序列非平稳。

如表2所示,百度指数在“报废汽车”、“过路费”、“二手货车”和“$oil0$”进行ADF检验,结果“过路费”和“二手货车”的显著性均小于5%的水平条件,即拒绝原假设,存在平稳分布,“报废汽车”和“$oil0$”的时间序列显著性均大于5%的水平条件,接受原假设,不存在平稳分布。

表2　ADF平稳性检验

变量	报废汽车	过路费	二手货车	oil0	运价
ADF	－2.418	－8.882	－4.084	－2.295	－2.457
P值	0.402	0.01	0.01	0.454	0.126

“报废汽车”、“oil0”和运价的时间序列经检验虽然是自身非平稳,但其与“过路费”和“二手货车”时间序列的某种线性组合却可能是长期均衡关系。这种线性组合反映了变量之间的长期稳定的比例关系,称为协整(cointegration)关系。本文使用Johansen提出的关于系数矩阵的协整似然比(LR)检验方法对多变量时间序列进行协整检验。

对于p阶的VAR,将该VAR转换成下列形式的向量误差修正模型(VECM):

$$\Delta\boldsymbol{y}_t = \prod\boldsymbol{y}_{t-1} + \sum_{i=1}^{p-1}\Gamma_i\Delta\boldsymbol{y}_{t-i} + B\boldsymbol{x}_t + \boldsymbol{\varepsilon}_t \quad (5)$$

其中,

$$\prod = \sum_{i=1}^{p-1}A_i - I,\quad \Gamma_i = -\sum_{j=i+1}^{p}A_j \quad (6)$$

如果rank(Π)＝0则$\boldsymbol{y}_t$所含n个变量都是单位根过程,此时因为不存在这n个变量的线性组合是平稳的,所以变量间不是协整的;如果rank(Π)＝n,则模型(6)是差分方程的收敛系统,因此所有变量都是平稳的;而0＜rank(Π)＜n的情况才是协整分析所关心的情况。

表3检验结果表明,在协整检验中,统计量小于1%水平下的临界值,因而有一个原假设被拒接,相应地存在一个协整方程拒绝$R\leqslant 3$的假设。协整检验结果证明百度指数的“二手货车”、“报废汽车”、“过路费”、“oil0”、运价之间存在长期稳定的均衡关系。

表3　Johansen协整检验结果

假设	test	10%	5%	1%
$R\leqslant 3$	5.00	10.49	12.25	16.26
$R\leqslant 2$	77.35	22.76	25.32	30.45
$R\leqslant 1$	238.48	39.06	42.44	48.45

2.4　预测模型拟合度分析

在对相关影响因素的时间序列进行检测与协整检验后,在模型进行训练之前,要对影响因素以及公路运价指数进行标准化。采用Z-score标准归一化方法,经该方法处理后的数据可呈现标准正态分布,函数表达式为$f(x)=(x-\mu)/\sigma$,其中μ表示样本数据的均值,σ表示样本数据的标准差。

确定模型的参数,确定输入隐层数和迭代次数分别为5和1000。在该网络模型激活函数的选择部分,以Simiod函数作为实验的激活函数,并选择接收ELM神经网络输出为最小的激活函数形式。对于ELM神经网络模型结构,经反复检验,选取最优参数为4层隐藏层,每层为1 604个神经元。考虑到模型的预测效果,将数据划分成两个部分:80%的数据集用作训练,而20%的数据集用作测试。对于选择损失函数来确定误差的计算方式,采用均方根误差$RMSE$(root mean squard error)以及平均绝对百分比误差$MAPE$(mean absolute percent error)指标来进行模型误差的度量。

1)均方根误差:

$$RMSE = \sqrt{\frac{1}{n}\sum_{t=1}^{T}(y_t - \hat{y}_t)^2} \quad (7)$$

(C)1994-2023 China Academic Journal Electronic Publishing House. All rights reserved.　http://www.cnki.net

2)平均绝对百分比误差：

$$MAPE = \frac{1}{n}\sum_{t=1}^{T}\left|\frac{y_t - \hat{y}_t}{y_t}\right| \quad (8)$$

在模型训练结束后，为了更准确地得到预测值，引入滚动窗口得到模型的最佳拟合效果。图 4、图 5 显示了经时域优化的模型精度，可知模型精度 MAPE 与 RSME 均随时域优化表现趋同，首先模型精度在滚动步距到达 62 d 以前精度不断提高，MAPE 与 RSME 分别由未优化的 2.50％和 35.09 提高到第 62 d 的 1.85％与 25.17。在 62 d 后，模型预测精度逐渐下降并反复波动。

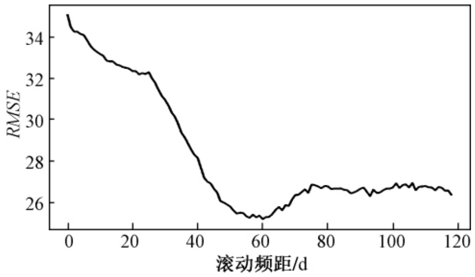

图 4　时域优化的 RMSE

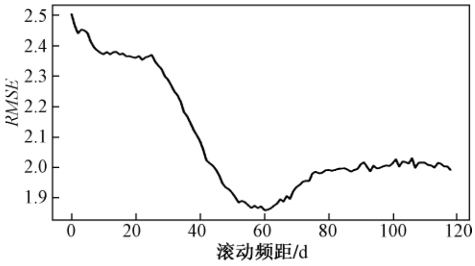

图 5　时域优化后的 MAPE

从表 4 中可以看出，样本内预测结果相对误差最小为 0.02％，最大为 5.80％。说明对于公路物流运价指数相对复杂的序列而言，可以使用 ELM 预测模型在精度和效率上实现提前预测，使得结果更加接近实际情况，具有科学的指导意义。

表 4　ELM 模型样本内预测结果比较

周次	实际值	预测值	相对误差/％
1	1 020.18	991.03	2.86
2	1 027.10	1 043.13	1.56
3	1 043.90	1 043.65	0.02
4	1 077.10	1 083.20	0.57
5	967.39	986.14	1.94
6	997.40	1 023.32	2.60
7	999.20	1 016.66	1.75
8	985.53	988.70	0.32
9	987.28	992.48	0.53
10	1 011.30	1 016.64	0.53

ELM 模型各预测值与实际值的数据对比结果如图 6 所示。预测结果具备良好的跟随性。综上可知滚动步距为 62 d 的 ELM 模型在预测公路运价指数时有良好的精度，能够预测公路运输市场的行情波动变化。

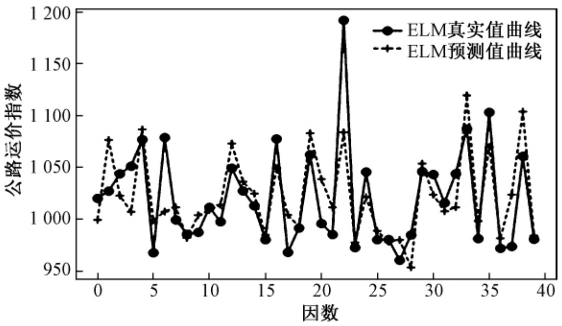

图 6　ELM 模型结果比较

3　结论

公路运价指数除受过路费、油价、车辆之久和经济环境因素影响外，其本身还与政策变化、节假日车辆变化等因素有关。针对公路运价指数统计指标复杂导致的成本高昂与发布时间滞后问题，提出一种基于百度指数的滚动极限学习机预测方法。该方法首先提取具有表征性的日百度指数，根据 ADF 平稳性检验与协整关系检验确定百度指数与公路运价指数间的长期均衡关系；然后利用滚动窗口对百度指数与公路运价指数间的结构关系进行优化，最后用极限学习机模型计算出不同时期的预测值。相比传统的计量方法，运用大数据和人工智能预测公路运价指数可以对有力支撑公路运输市场精确计量，有效预测提前预示了公路运输市场的发展变化，为科学合理决策助力。

参考文献

[1] 普华永道，中国交通报. 中国公路货运行业智慧安全白皮书［EB/OL］.［2020-05-14］. https://www.pwccn.com/zh/industries/automotive/publications/white-paper-intelligent-safety-china-road-freight.html.

[2] 中国物流与采购联合会. 公路物流运价指数打造成经济“晴雨表”［EB/OL］.（2015-08-03）. http://www.chinawuliu.com.cn/lhhzq/201508/03/303865.shtml.

[3] 顾敬岩，吴群琪. 我国公路货运市场价格的演进趋势、问题及对策［J］. 交通运输系统工程与信息，2013，13(6)：191－197.

[4] 戴宏. 我国公路货运物流服务定价机制研究［J］. 价格理论与实践，2016(12)：74－76.

[5] 胡欣. 基于物流车辆的数据挖掘和公路物流指数研究［D］. 武汉：武汉理工大学，2016.

(C)1994-2023 China Academic Journal Electronic Publishing House. All rights reserved.　http://www.cnki.net

[6] 彭建良,丁怡越,左晓琴.中国公路物流运价指数预测研究——基于 ARIMA-Adam-LSTM 模型的分析[J].价格理论与实践,2019(6):99－102.

[7] 中国互联网络信息中心.第 44 次中国互联网络发展状况统计报告[EB/OL].(2019－08－30).http://www.cnnic.net.cn/hlwfzyj/hlwxzbg/hlwtjbg/201908/t20190830_70800.htm.

[8] 张崇,吕本富,彭赓,等.网络搜索数据与 CPI 的相关性研究[J].管理科学学报,2012,15(7):50－59.

[9] GINSBERG JEREMY, MOHEBBI MATTHEW H, PATEL RAJAN S, et al. Detecting influenza epidemics using search engine query data[J]. Nature,2009,457:1012－1014.

[10] WU LYNN, BRYNJOLFSSON ERIK. The future of prediction: how google searches foreshadow housing prices and quantities[C]//ICIS 2009 Proceedings. 2009: 147－148.

[11] TANG J, DENG C, HUANG G B, et al. Compressed-domain ship detection on spaceborne optical image using deep neural network and extreme learning machine[J]. IEEE Transactions on Geoscience and Remote Sening,2014,53(3):1174－1185.

[12] HUANG G B, ZHU Q Y, SIEW C K. Extreme learning machine: a new learning scheme of feedforward neural networks[C]//IEEE International Joint Conference on Neural Networks. IEEE,2005,2(2):985－990.

[13] HUANG G B, ZHU Q Y, SIEW C K. Extreme learning machine: theory and applications[J]. Neurocomputing, 2005:70(1):489－501.

[14] HUANG G B. Learning capability and stor-age capacity of two-hidden-layer feedforward networks[J]. IEEE Transactions on Neural Net-Works,2003,14(2):274－281.

[15] HUANG G B, BABRI H A. Upper bounds on the number of hidden neurons in feed forward neworks with arbitrary bounded nonlinear activetion functions[J]. IEEE Transactions on Neural Neworks,1998,9(1):224－229.

[16] 张锡平,吴甦,林亨,徐超,王剑钊,李勇,路晖.2005 中国物流总成本研究[J].中国物流与采购,2006(4):24－29.

[17] HUANG G B, CHEN LEI, SIEW C K. Universal approximation using incremental constructive feedforward networks with random hidden nodes[J]. IEEE Transactions on Neural Neworks,2006,17(4):879－892.

[18] 刘国宝,张洁.基于改进滚动时域优化策略的动态调度方法[J].机械工程学报,2013,49(14):182－190.

[19] ASKITAS N, ZIMMERMANN K F. Google econometrics and unemployment forecasting[J]. Discussion Papers of Diw Berlin,2009,55(2):107－120.

Rolling Extreme Learning Machine Forecast of Highway Freight Index Based on Baidu Index

ZHU Xi[1], LAI Ying-liang[1], DUAN Yu-tong[2]

(1. Faculty of Civil Engineering and Mechanics, Kunming University of Science and Technology, Kunming 650093 China;
2. School of History and Archives, Yunnan University, Kunming 650093, China)

Abstract: Highway transportation price index is a measure of the fluctuation of highway transportation market, which has an important predictive function for China's highway transportation industry. The extreme learning machine (ELM) neural network model is used to predict the highway freight index quickly and at low cost. Using the Person correlation coefficient of Baidu index and highway freight index to determine the impact of component on the highway freight index, and then the ADF stationarity test and Johansen cointegration test were used to construct the input sequence, and finally the window scroll was used to optimize the input variables. The predicted value is output in the ELM model. The results show that the ELM model based on the rolling window improved average absolute percentage error and root mean square error compared with the single ELM model. The prediction results are consistent with the index fluctuations, which can provide a decision reference for the direction of the highway freight index.

Key words: highway transportation of economic; highway transportation price index; extreme learning machine; prediction

(C)1994-2023 China Academic Journal Electronic Publishing House. All rights reserved. http://www.cnki.net
