# Supplementary material for: Granting analysis of the credit financing of the platform-type highway transportation supply chain
Source: PLoS One. 2024 Jul 10;19(7):e0303807. doi: 10.1371/journal.pone.0303807 (PMC11236115; doi:10.1371/journal.pone.0303807)
Supplement: S2 File — (PDF) [file pone.0303807.s003.pdf]

基于互惠动机的平台型供应链金融利益权衡机制

占永志 陈金龙 邹小红
(华侨大学 工商管理学院 福建 泉州 362021)

摘 要: 以 Nafin 反向保理平台中的核心企业与供应商之间的利益协调问题为背景,以 DK 序贯互惠博弈模型为基础,构建了平台型供应链金融中基于供应商互惠的核心企业提供融资支持与供应商接受核心企业延长信用期要求的两人两阶段序贯互惠博弈模型,分析了供应商接受核心企业延长信用期要求的互惠心理条件和物质效用条件,为核心企业合理进行利益权衡提供了直接决策依据。

关键词: 平台型供应链金融; 反向保理; 互惠动机; 序贯互惠博弈; 利益权衡
中图分类号: N949 **文献标识码:** A **文章编号:** 1005-6408(2018)02-0131-06

引言

墨西哥国家财政发展银行于 1934 年创建的 Nafin 平台是一个提供在线反向保理服务的供应链金融平台。Nafin 平台主要功能是在核心企业与其中小供应商之间创建关系链,为供应商提供应收账款反向保理融资服务[1]。

Nafin 平台向供应商收取融资额 0.05% 的手续费。金融机构一般向供应商提供应收账款账面价值 70% 的融资额,剩余部分在收到核心企业的全额货款后再支付给供应商,并从中扣除贷款利息和平台手续费[2]。

供应商的应收账款来源于核心企业,应收账款反向保理离不开核心企业的支持。核心企业因支持供应商反向保理融资而获得有利谈判条件,从而向供应商提出相应利益要求。

在实际交易谈判中,不同核心企业提出的利益要求不尽相同。本文拟选择核心企业要求供应商延长应收账款信用期的情形进行分析,且这种情形在 Nafin 平台比较普遍。核心企业要求延长信用期对供应商物质利益产生直接影响,需要合理决策。以往人们仅从单纯的物质利益角度去分析双方的利益均衡策略,然而,随着管理领域互惠理论的研究不断发展,人们逐渐认识到互惠动机对合作方利益均衡策略的影响。因此,核心企业要做出最佳决策,除了供应商的物质利益外还需考虑其互惠动机。所谓互惠动机是指行为人具有报答他人善意行为和报复他人敌意行为的动机,即使实施报答和报复行为会导致一定的额外成本。核心企业对供应商融资提供支持,具有互惠动机的供应商会加以回报; 否则,核心企业不支持,供应商则会采取相应的不友好甚至报复行为。核心企业的支持使供应商感受到其友好,获得心理效用,从而也表现出更积极的合作态度; 同时,核心企业的示好行为,也希望供应商能作出回报,这正是经济学互惠理论的基本要义。

关于供应链金融参与主体之间行为博弈,许多学者从不同角度进行了研究[3-5],但其共同点是基于理性的“经济人”视角,忽视了行为主体的互惠动机。

Rabin[6] 较早分析了合作方博弈中的互惠心理因素,在此基础上,Dufwenberg 和 Kirchsteiger[7] 提出了序贯互惠博弈模型。从目前看,互惠动机理论已被引入委托代理[8]、供应链合作[9]、员工管理[10] 等方面。

本文以 Nafin 反向保理平台中的核心企业与供

*
收稿日期: 2016-12-08
基金项目: 国家自然科学基金(71571074)
作者简介: 占永志(1976-),男,江西景德镇人,华侨大学工商管理学院企业管理博士生,研究方向: 财务管理;
陈金龙(1965-),男,福建龙海人,管理学博士,华侨大学工商管理学院教授,博士生导师,研究方向: 金融工程与金融管理。
E-mail: zyzhi760307@163.com

(C)1994-2023 China Academic Journal Electronic Publishing House. All rights reserved. http://www.cnki.net

应商之间的利益协调问题为背景，主要从核心企业视角分析平台型供应链金融的利益权衡机制。论文的主要创新在于分析核心企业利益权衡策略时同时考虑了供应商的互惠效用和物质效用，弥补了已有文献仅考虑物质效用的局限。

1 DK 序贯互惠模型

Dufwenberg 定义了序贯互惠均衡(sequential reciprocity equilibrium，SRE)的概念，并证明每个具有互惠激励因素的博弈中存在一个 SRE。Dufwenberg 和 Kirchsteiger(2004)互惠效用函数由以下三部分组成。

1.1 公平支付

$$\pi_j^{ei}((b_{ij})_{j\neq i}) = \frac{1}{2}\left[\max_{ai\in Ai}\{\pi_j(a_i(t),(b_{ij}(t))_{j\neq i}) \mid a_i \in A_i\} + \min_{ai\in Ai}\{\pi j(a_i(t),(b_{ij}(t))_{j\neq i}) \mid a_i \in E_i\}\right] \quad (1)$$

其中，$\pi_j^{ei}((b_{ij})j\neq i)$ 是参与者 j 关于参与者 i 的公平支付，其被定义为给定参与者 j 的策略 b_{ij}，参与者 i 的所有策略 ai 给参与者 j 带来的最大收益与最小收益的平均值，E_i 表示 i 的可选策略集。$E_i=\{a_i\in A_i \mid$ 不存在 $a'\in A_i$，$s.t.$ 对所有的 $t\in R$，$(a_i)j\neq i\in\prod_{j\neq i}A_j$，$s\in N$ 满足 $\pi_k(a'_i(t),(a_j(t))_{j\neq i})\geqslant\pi k(a_i(t),(a_j(t))_{j\neq i})$，对有些$(t,(a_j)_{j\neq i},s)$严格不等式成立$\}$。$i,j,s\in N$ 表示三个参与方，$N=\{1,2,\cdots,n\}$ 为参与方集合，R 为参与方历史博弈节点集，t 为某节点，Ai 是参与方 i 的策略集，a_i，a_j 表示参与方 i 和 j 的一个策略，$b_{ij}\in B_{ij}$，$B_{ij}=A_i$ 是 i 对 j 的策略的信念(一阶信念)，$c_{ijs}\in C_{ijs}$，$C_{ijs}=B_{js}=A_s$ 是 i 对 j 对 s 信念的信念(二阶信念)，$a_i(t)$ 表示 i 在节点 t 采取的策略。

1.2 友善函数

参与方 i 对参与方 j 的友善函数 k_{ij} 表示为:

$$k_{ij}(a_j(t),(b_{ij}(t))j\neq i) = \pi_i(b_{ij}(t),(c_{iji}(t))_{i\neq j}) - \pi_j^{ei}((c_{iji}(t))_{i\neq j}) \quad (2)$$

这意味着，参与者 i 对参与者 j 的善意被定义为一个差值，即参与者 i 试图给予参与者 j 的期望物质支付 $\pi_j(\cdot)$ 与一个公平的期望物质支付 $\pi_j^{ei}(\cdot)$ 的差值。

1.3 感知善意函数 λ_{iji}

参与者 i 所感知到的参与者 j 善意的信念由下式给出:

$$\lambda_{iji}(b_{ij}(t),(c_{iji}(t))_{i\neq j}) = \pi i(b_{ij}(t),(c_{iji}(t))_{i\neq j}) - \pi_j^{ei}((c_{iji}(t))_{i\neq j}) \quad (3)$$

上式与友善度的定义类似，感知到的善意也是一个差值，即参与者 i 认为他所获得的期望物质支付 $\pi i(\cdot)$ 与一个公平的期望物质支付 $\pi_j^{ei}(\cdot)$ 的差值。

1.4 效用函数 U_i

参与者 i 的效用函数为

$$U_i(a_i,b_{ij},c_{iji})_{j\neq i} = \pi_i(a_i,(b_{ij})_j\neq i) + \sum_{j\neq i}(Y_{ij}\cdot k_{ij}(a_i,(b_{ij})_{j\neq i})\cdot\lambda_{iji}(b_{ij},(c_{iji})_{j\neq i})) \quad (4)$$

这里，对于每一个 $j\neq i$，Y_{ij} 是一个非负互惠敏感系数。在上式中，等式后面第一部分 $\pi_i(\cdot)$ 与第二部分 $\sum_{j\neq i}Y_{ij}\cdot k_{ij}(\cdot)\lambda_{iji}(\cdot)$ 分别代表了参与者 i 的期望物质支付与期望心理支付。参与者 i 的心理支付包括了：参与者 i 对参与者 j 善意(或不善)的敏感度 Y_{ij}，参与者 i 对参与者 j 的善意 $k_{ij}(\cdot)$ 以及参与者 j 所感知到的参与者 j 对他的善意 λ_{iji}。

1.5 序贯互惠均衡(SRE)

对于所有的参与者 $i\in N$ 以及每一个决策点 $t\in R$ 来说，当以下成立时:

(1) a_i^* $\mathrm{argmax}_{ai\in Ai}U_i(a_i,b_{ij},c_{iji})_{j\neq i}$;

(2) $b_{ij}=a_j^*$ 对所有的 $j\neq i$;

(3) $c_{iji}=a_i^*$ 对所有的 $j\neq i$。

则策略组合 $a^*=(a_i^*)i\in N$ 是一个连续互惠均衡(SRE)。

2 基于供应商互惠的两阶段序贯互惠模型构建与分析

2.1 模型的基本设定

假设 1：供应商单次保理的应收账款平均账面金额为 L，金融机构提供的融资比例为 α，则供应商在一次反向保理中实际融资额为 αL。

假设 2：在原有交易中，供应商对核心企业提供的信用期为 T_0，核心企业要求延长的信用期为 T。

假设 3：供应商将所得资金用于生产投资，其投资平均收益率为 R_e，融资月利率为 R_b，平台按融资额向供应商收取的手续费率为 R_f。

假设 4：核心企业平均投资收益率为 R_c，同时，核心企业提供支持需要付出一定的成本，在供应商的一次保理融资中，核心企业需支付的平均成本为 C_c。

2.2 完全理性假设下的序贯互惠博弈分析

博弈双方为核心企业 j 和供应商 i，且假设为完全理性，即双方都追求自身物质效用的最大化。供应商的策略集为：$t\in\{y,n\}$，，$t=y$ 表示接受核心企业延长信用期的要求；$t=n$ 表示拒绝延长信用期。

(C)1994-2023 China Academic Journal Electronic Publishing House. All rights reserved. http://www.cnki.net

核心企业的策略集为：$t' \in \{y, n\}$，$t' = t$ 表示核心企业支持供应商的应收账款反向保理融资；$t' = n$ 则表示核心企业不予支持。博弈分为两个阶段，第一阶段，核心企业选择支持或不支持，第二阶段供应商选择接受或拒绝核心企业要求。双方博弈过程用博弈树表示，见图 1。

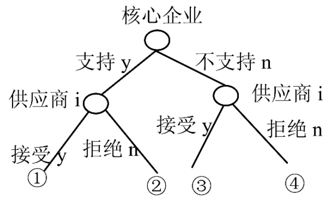

图 1　核心企业与供应商的序贯博弈树

根据模型假设和博弈树可得核心企业与供应商的收益矩阵，如表 1 所示。

表 1　核心企业——供应商收益矩阵

博弈策略	核心企业 j 收益	供应商 i 收益
①(y, y)	$LTRc - Cc$	$aLT_0(R_e - R_b) - aLR_f - aLTR_b - (1 - a) LTR_e$
②(y, n)	$-C_c$	$aLT_0(R_e - R_b) - aLR_f$
③(n, y)	LTR_c	$-LTR_e$
④(y, n)	0	0

在博弈树中，$aLT_0(R_e - R_b)$ 为供应商将应收账款通过反向保理平台融资后，将所得资金再投资在 T_0（原信用期）期间所取得的净收益，aLR_f 是供应商向平台交纳的手续费，$aLTR_b$ 是供应商在 T（延长的信用期）期间内需支付的银行利息，$(1 - a) LTR_e$ 是供应商保理的应收帐款余款在 T（延长的信用期）期间内应取得的投资收益，但金融机构需要在收到核心企业货款，即在 T（延长的信用期）到期才将余款结清，因而供应商失去了余款的投资收益，意味着供应商给核心企业延长信用承担了这部分损失。从博弈树可以看出，核心企业可以选择两种策略：①支持供应商的应收账款保理融资；②不支持供应商的应收账款保理融资。供应商同样可以选择两种策略：① 接受核心企业要求，延长信用期；② 拒绝核心企业要求，不延长信用期。

如果供应商不具有互惠动机，即 $Y_{ij} = 0$，由式(4)可知，其互惠心理效用等于 0，则供应商会从物质收益最大化出发进行策略选择。因此，根据核心企业和供应商在不同策略组合下的物质收益函数（如图 1 所示），用逆向归纳法可推知，无论核心企业支持或不支持，供应商选择拒绝策略下的物质收益都大于选择接受策略下的物质收益，因此，供应商都会选择拒绝策略，从而核心企业也会选择不支持策略，于是可以得到博弈双方的最优行为策略组合：核心企业对供应商的应收账款保理不提供支持，供应商拒绝核心企业延长信用期的要求。在这种情况下核心企业和供应商的合作关系将无法形成。不失一般性，如果供应商的互惠心理效用很小，则他们的行为策略仍然不变。因而得到本文的第一个研究结论：当供应商具有完全理性的时候，核心企业将不支持供应商的应收账款反向保理融资，供应商拒绝核心企业延长信用期要求，双方无法建立互惠合作关系。

2.3　考虑供应商互惠动机的博弈模型

由式(4)互惠心理效用函数可知，当 $Y_{ij} = 0$ 时，$U_i(a_i, b_{ij}, c_{iji}) = \pi_i(a_i, b_{ij})$，表示企业只关注物质效用，这是不考虑互惠动机的理性人假设下的效用函数，这种情形在前面已作分析。

如果供应商具有互惠动机（$Y_{ij} > 0$），当供应商认为核心企业对其友善（$Y_{ij} > 0$）时，供应商会采取友善行为（$\lambda_{iji} > 0$）以获得效用最大化；反之，供应商认为核心企业对其不友善（$\lambda_{iji} < 0$）时，它会采取不友善行为（$k_{ij} < 0$），以避免效用损失。

所以，本文只讨论 $Y_{ij} > 0$ 情形下的博弈行为。

2.3.1　核心企业选择不支持策略时的均衡分析

当核心企业选择不提供融资支持时，供应商选择拒绝策略的物质收益 $\pi_i(n \mid t = n) = 0$，大于其选择接受策略时的物质收益 $\pi_i(y \mid t = n) = -LTR_e$。此时，供应商认为核心企业是不友善的（$\lambda_{iji} < 0$），根据式(4)效用函数，供应商选择拒绝策略（$k_{iji} < 0$）时其效用会增加，即 $U_i(n \mid t = n) > U_i(y \mid t = n)$，可见，供应商的物质收益和互惠收益都会比其选择接受策略时高；并且，供应商所获得的效用高于理性人假设条件下的效用，即 $U_i(n \mid t = n) > 0$，从而供应商选择拒绝策略的动机更为强烈。

命题 2 在考虑供应商互惠动机后，如果核心企业选择不支持策略，则供应商也会选择拒绝策略，而且其选择拒绝策略的动机比理性人假设条件下更为强烈，双方更加无法建立互惠合作关系。

2.3.2　核心企业选择支持策略时的均衡分析

(1) 供应商对核心企业友善度 k_{ij} 的确定

由式(2)可知，供应商对核心企业友善函数 k_{ij} 等于其所选择策略对核心企业产生的期望物质支付 $\pi_j(\cdot)$ 与一个公平的期望物质支付 $\pi_j^{ei}(\cdot)$ 的差值，因此，当核心企业选择支持策略时，由式(1)可

(C)1994-2023 China Academic Journal Electronic Publishing House. All rights reserved.　http://www.cnki.net

知，供应商给核心企业带来的平均物质收益为

$$\pi_j^{ei} = \frac{1}{2}LTR_c - C_c \tag{5}$$

1）当供应商选择接受策略时，其对核心企业的友善度为

$$k_{ij}(y \mid t = y) = LTR_c - C_c - \frac{1}{2}LTR_c + C_c = \frac{1}{2}LTR_c \tag{6}$$

2）当供应商选择拒绝策略时，其对核心企业的友善度为

$$k_{ij}(n \mid t = y) = -C_c - \frac{1}{2}LTR_c + C_c = -\frac{1}{2}LTR_c \tag{7}$$

由假设条件可知，$L, T, R_c > 0$ 所以有：$k_{ij}(y \mid t = y) > 0$，$k_{ij}(n \mid t = y) < 0$。

（2）供应商感知到的核心企业的友善度 $\lambda_{iji}(b_{ij}, c_{iji})$ 的确定

由式（3）可知，$\lambda_{iji}(b_{ij}, c_{iji})$ 为供应商对核心企业策略所带来的支付的期望与供应商认为核心企业可以带给它的平均支付之差。用 $p(0 \leqslant p \leqslant 1)$ 表示供应商选择接受策略的概率，则其选择拒绝策略的概率为 $1-p$。用 $p'(0 \leqslant p' \leqslant 1)$ 表示核心企业对供应商选择接受策略的概率的期望，也即对 p 的期望。用 $p''(0 \leqslant p'' \leqslant 1)$ 表示供应商对核心企业对供应商选择接受策略的概率的期望 p' 的期望，也即供应商对核心企业认为自己多大可能性会选择接受策略的判断。结合命题2，供应商认为核心企业可以带来的平均收益为

$$\pi_i^{ej} = \frac{1}{2}\{p''[aLT_0(R_e - R_b) - aLR_f - aLTR_b - (1-a)LTR_e] + (1-p'')[aLT_0(R_e - R_b) - aLR_f]\} \tag{8}$$

当核心企业选择支持策略时，供应商感知到的核心企业的友善度为

$$\lambda_{iji}(t = y) = p[aLT_0(R_e - R_b) - aLR_f - aLTR_b - (1-a)LTR_e] + (1-p)[aLT_0(R_e - R_b) - aLR_f] - \pi_i^{ej} \tag{9}$$

（3）序贯互惠均衡的求解

根据 Dufwenberg 和 Kirchsteiger（2004）关于序贯互惠均衡的理论，当达到序贯互惠均衡（SRE）时，每个博弈方都根据其信念做出了最优决策，所有高阶信念都与实际行为相符，即有

$$p = p' = p'' \tag{10}$$

由式（4）效用函数可知，当核心企业选择支持策略时，供应商选择接受策略下的效用函数为

$$U_i(y \mid t = y) = \pi_i(y \mid t = y) + Y_{ij} \cdot K_{ij}(y \mid t = y) \cdot \lambda_{iji}(t = y) \tag{11}$$

将式（6）、（8）、（9）、（10）代入式（11）得

$$U_i(y \mid t = y) = aLT_0(R_e - R_b) - aLR_f - aLTR_b - (1-a)LTR_e + \frac{1}{4}Y_{ij}LTR_c[-paLR_f - paLTR_b - p(1-a)LTR_e + aLT_0(R_e - R_b)] \tag{12}$$

同理，当核心企业选择支持策略，供应商选择拒绝策略时其效用函数为

$$U_i(n \mid t = y) = \pi_i(n \mid t = y) + Y_{ij} \cdot K_{ij}(n \mid t = y) \cdot \lambda_{iji}(t = y) \tag{13}$$

将式（7）、（8）、（9）、（10）代入式（13）得

$$U_i(n \mid t = y) = aLT_0(R_e - Rb) - aLR_f - \frac{1}{4}Y_{ij}LTR_c[-paLR_f - paLTR_b - p(1-a)LTR_e + aLT_0(R_e - R_b)] \tag{14}$$

1）当核心企业选择支持策略时，如果供应商选择接受策略比选择拒绝策略的效用更高，即有

$$U_i(y \mid t = y) > U_i(n \mid t = y) \tag{15}$$

此时，供应商选择接受策略，则有

$$p = p' = p'' \tag{16}$$

将式（12）、（14）、（16）代入式（15）得

$$Y_{ij} > \frac{2aR_b + 2(1-a)R_e}{R_c[-aLR_f - aLTR_b - (1-a)LTR_e + aLT_0(R_e - R_b)]} \tag{17}$$

令 $Y_{ij}^h = \dfrac{2aR_b + 2(1-a)R_e}{R_c[-aLR_f - aLTR_b - (1-a)LTR_e + aLT_0(R_e - R_b)]}$，由式（17）可知，当供应商互惠敏感系数 $Y_{ij} > 0$ 时，必须满足

$$aLT_0(R_e - R_b) > aLR_f + aLTR_b + (1-a)LTR_e \tag{18}$$

由式（18）可知，在核心企业选择支持时，如果供应商通过反向保理融资提前取得资金所能获得的额外净收益大于上述手续费、利息费用和损失之和，则 $Y_{ij} > Y_{ij}^h$，此时，虽然供应商选择接受策略会损失物质收益（其损失物质收益为 $aLR_f + aLTR_b + (1-a)LTR_e$，见博弈树），但由式（12）、（14）、（15）可知，供应商的互惠收益增加量超过其物质收益的减少量，从而总效用增加。因此，核心企业选择支持策略

(C)1994-2023 China Academic Journal Electronic Publishing House. All rights reserved. http://www.cnki.net

的情况下供应商会选择接受策略，达到帕累托均衡。

2）当核心企业选择支持策略时，如果供应商选择拒绝策略比选择接受策略时的互惠效用更高，即有

$$U_i(y \mid t = y) < U_i(n \mid t = y) \quad (19)$$

此时，供应商会选择拒绝策略，则有

$$p = p' = p'' \quad (20)$$

将(12)、(14)、(20)式代入(19)式，解得

$$Y_{ij} < \frac{2aR_b + 2(1-a)R_e}{R_c aLT_0(R_e - R_b)} \quad (21)$$

令 $Y_{ij}^l = \frac{2aR_b + 2(1-a)R_e}{R_c aLT_0(R_e - R_b)}$，式(21)表明，核心企业选择支持策略的情形下，当供应商的互惠动机 $Y_{ij} < Y_{ij}^l$ 的时候，由式(12)、(14)、(19)可知，其选择接受策略的互惠收益增加量低于其物质收益的损失量，总效用减少，因而供应商会选择拒绝策略。

3）当核心企业选择支持策略时，如果供应商选择接受策略和选择拒绝策略互惠效用相等，即有

$$Y_{ij}^l < Y_{ij} < Y_{ij}^h \quad U_i(y \mid t = y) = U_i(n \mid t = y) \quad (22)$$

将(12)、(14)式代入(22)式，解得

$$p = \frac{aT_0(R_e - R_b)}{aR_f + aTR_b + (1-a)TR_e} - \frac{2aR_b + 2(1-a)R_e}{Y_{ij}R_c[aLR_f + aLR_b + (1-a)LTR_e]} \quad (23)$$

由式(23)易知 p 是 Y_{ij} 的单调增函数，而 $p(Y_{ij} = Y_{ij}^l) = 0$，$p(Y_{ij} = Y_{ij}^h) = 1$，所以当 $Y_{ij}^l < Y_{ij} < Y_{ij}^h$ 时有 $0 < p < 1$。由此可知，由于 p 是 Y_{ij} 的单调增函数，供应商的互惠动机越强，即 Y_{ij} 越大，则其选择接受策略的概率越大。

命题 3 如果供应商具有互惠动机，核心企业选择支持策略，存在以下三种序贯互惠均衡：

1）当 $Y_{ij} > Y_{ij}^h$，如果满足 $aLT_0(R_e - R_b) > aLR_f + aLTR_b + (1-a)LTR_e$，即供应商通过反向保理融资提前取得资金，该资金在 T_0（原信用期）期间用于投资所能获得的额外净收益大于平台手续费、供应商在 T（延长的信用期）期间承担的利息费用与应收账款的余款在 T（延长的信用期）期间失去投资机会而产生的损失之和，供应商的总效用增加，从而选择接受策略。

2）当 $Y_{ij} < Y_{ij}^l$，供应商选择接受策略时互惠收益增加量低于其物质收益的损失量，总效用减少，因而供应商会选择拒绝策略。

③ 当 $Y_{ij}^l < Y_{ij} < Y_{ij}^h$，供应商以一定概率选择接受策略，其概率随 Y_{ij} 的增加而增加。

结语

本文以 DK 序贯互惠博弈模型为基础，同时考虑供应链金融平台中供应商的物质效用和互惠心理效用，构建了基于供应商互惠的核心企业提供融资支持与供应商接受核心企业延长信用期要求的两人两阶段序贯互惠博弈模型，并通过模型分析得出三条研究命题。命题指出了供应商接受核心企业延长信用期要求的互惠心理条件（互惠敏感系数 Y_{ij} 的取值范围）和物质效用条件，为核心企业合理确定信用期的延长期限、进行利益权衡提供了直接决策依据。

尽管本文获得了一些有意义的结论，但也存在一些局限：(1)从论文的博弈分析过程不难发现，互惠敏感系数 Y_{ij} 受物质收益的影响，但二者之间有没有确定的关系？能否用物质收益作为变量求解互惠敏感系数？如能求解，将对核心企业的利益决策提供直接依据，对此，论文并没有展开研究。(2)本文仅仅把序贯互惠均衡应用到了两个参与者之间，如何引入下游经销商、商业银行等参与主体、研究更多主体在供应链金融平台中的互惠博弈均衡，将是下一步需要解决的问题。

参考文献

[1] Kasper V D V，Reindorp M，Fransoo J C. Improving Service Levels through Reverse Factoring [J]. Social Science Elect-ronic Publishing，2015，6(2)：14-21.

[2] Bakker M H R，Udell G F，Klapper L F. Financing Small and Medium-Size Enterprises with Factoring：Global Gro-wth and Its Potential in Eastern Europe [J]. Social Science Electronic Publishing，2004，7(3)：46-52.

[3] John A. Buzacott&Rachel Q. Zhang. Inventory Management with Asset Based Financing [J]. Management Science，2004，50(9)：1274-1292.

[4] 吕永卫，孙西生. 基于演化博弈理论的供应链系统长期稳定性分析 [J]. 系统科学学报，2013，21(2)：64-66.

[5] 胡盛强，张毕西，刘绘珍，等. 基于多方博弈的二级网状供应链合作及利润分配研究 [J]. 系统科学学报，2012，20(2)：48-51.

[6] Rabin M. Incorporating Fairness into Game Theory and Economics [C]. University of California at Berkeley，1992，35(7)：1281-1302.

[7] Dufwenberg M，Kirchsteiger G. A theory of Sequential reciprocity [J]. Games & Economic Behavior，2004，47(2)：268-298.

[8] 蒲勇健，师伟. 基于利益冲突视角的互惠激励效应研究 [J]. 系统工程学报，2013，28(1)：28-37.

[9] 王迅，陈金贤. 供应链合作关系互惠机制与合约机制的演化分析

(C)1994-2023 China Academic Journal Electronic Publishing House. All rights reserved. http://www.cnki.net

[J]. 运筹与管理,2008,17(5):26-31.
[10] 钱峻峰,蒲勇健. 管理者——员工纵向信任行为分析——基于序贯互惠“囚徒困境”博弈模型[J]. 预测,2011,30(2):46-50.

Interest Balancing Mechanism of Platform - Led Supply Chain Finance Based on Reciprocal Motivation

ZHAN Yong-zhi CHEN Jin-long ZOU Xiao-hong

(*College of Business Administration*, *Huaqiao University*, *Quanzhou*, *Fujian* 362021, *China*)

Abstract: Under the background of interest coordination between the core enterprise and the supplier in the Nafin reverse factoring platform, based on the DK sequential reciprocity game model, this paper constructs a two stage sequential reciprocity model about the core enterprise which provides support for the supplier and the supplier accepts the core enterprise´s demand to extend the credit period of the Platform-Led supply chain finance based on the supplier´s reciprocity. And it analyzes the mutual psychological and material utility conditions for the supplier to accept the core enterprise´s demand to extend the credit period, provides a direct decision basis for the core enterprise to balance the interests with the supplier.

Key words: platform-led supply chain finance; reverse factoring; reciprocal motivation; sequential reciprocity game; interest balancing

(上接第79页)

Scientific Establishment of the Index System to Diagnose Large-Scale Construction Project Implement State

HOU Xue-liang WANG Yi

(*College of Economy and Management*, *North China Electric Power University*, *Beijing* 102206, *China*)

Abstract: The present diagnosis results to analyze macroscopic and meso-states of large-scale construction project can't meet construction project management requirements because of the lack of systematic connotations of the large-scale construction project implement state Index System. Through research on a lot of relative literatures and their problems, how to design and determine system grade of large-scale construction project has been analyzed on the guidance of systematic scientific theory, and a new frame of large-scale construction project implement state diagnosis index system is put forward according to real management requirements of large-scale construction project. The achievement is not only a new approach to solve this problem of systematic connotations, but also a new method to ensure the index system established by the way possessing systematicness, logicality as well as relativity.

Key words: systematic science; large-scale construction; index system; establishing method

(C)1994-2023 China Academic Journal Electronic Publishing House. All rights reserved. http://www.cnki.net
